# A nanobuffer reporter library for fine-scale imaging and perturbation of endocytic organelles

**DOI:** 10.1038/ncomms9524

**Published:** 2015-10-05

**Authors:** Chensu Wang, Yiguang Wang, Yang Li, Brian Bodemann, Tian Zhao, Xinpeng Ma, Gang Huang, Zeping Hu, Ralph J. DeBerardinis, Michael A. White, Jinming Gao

**Affiliations:** 1 Department of Pharmacology, Simmons Comprehensive Cancer Center, University of Texas Southwestern Medical Center, 5323 Harry Hines Boulevard, Dallas, Texas 75390, USA; 2 Department of Cell Biology, University of Texas Southwestern Medical Center, 5323 Harry Hines Boulevard, Dallas, Texas 75390, USA; 3 Children's Medical Center Research Institute, University of Texas Southwestern Medical Center, 5323 Harry Hines Boulevard, Dallas, Texas 75390, USA

## Abstract

Endosomes, lysosomes and related catabolic organelles are a dynamic continuum of vacuolar structures that impact a number of cell physiological processes such as protein/lipid metabolism, nutrient sensing and cell survival. Here we develop a library of ultra-pH-sensitive fluorescent nanoparticles with chemical properties that allow fine-scale, multiplexed, spatio-temporal perturbation and quantification of catabolic organelle maturation at single organelle resolution to support quantitative investigation of these processes in living cells. Deployment in cells allows quantification of the proton accumulation rate in endosomes; illumination of previously unrecognized regulatory mechanisms coupling pH transitions to endosomal coat protein exchange; discovery of distinct pH thresholds required for mTORC1 activation by free amino acids versus proteins; broad-scale characterization of the consequence of endosomal pH transitions on cellular metabolomic profiles; and functionalization of a context-specific metabolic vulnerability in lung cancer cells. Together, these biological applications indicate the robustness and adaptability of this nanotechnology-enabled ‘detection and perturbation' strategy.

## Abstract (web summary)

Endocytic organelles are a dynamic continuum of vacuolar structures that impact various cell physiological processes. Here, the authors report a library of ultra-pH sensitive fluorescent nanoparticles for the controlled perturbation and quantification of catabolic organelle maturation at organelle resolution.

## Graphical abstract

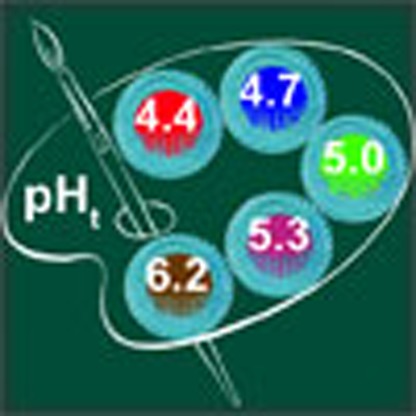

Endocytic organelles play an essential role in many cell physiological processes and are a primary site of cell–nanoparticle interactions. In cell biology, endosomes/lysosomes act as a nidus for signal transduction events that coordinate cell and tissue responses to nutrient availability and protein/lipid metabolism^1^,^2^,^3^. In drug and gene delivery, endosomes are the first intracellular organelles encountered after nanoparticle uptake by endocytosis^4^,^5^,^6^. Numerous nanocarriers are under development to achieve early endosomal release of therapeutic payloads and avoid lysosomal degradation^7^,^8^. A ubiquitous biological hallmark that affects all the above processes is the luminal pH of endocytic organelles^9^. For example, along the endocytic pathway, progressive acidification compartmentalizes ligand–receptor uncoupling (for example, low-density lipoprotein receptor) and activation of proteases for protein/lipid degradations into endosomes and lysosomes, respectively^1^,^2^. Most gene/siRNA delivery systems (for example, polyethyleneimines^10^) behave as a ‘proton sponge' to increase osmotic pressure of endosomes for enhanced cytosolic delivery of encapsulated cargo. Although there have been remarkable advances in the effectiveness of these delivery systems, little is known about how perturbations of endosomal/lysosomal pH by these nanoparticles may affect cell homeostasis. Reagents currently used to manipulate and study the acidification of endocytic organelles include lysosomotropic agents (for example, chloroquine (CQ) and NH_4_Cl), v-ATPase inhibitors (for example, bafilomycin A1) and ionophores (for example, nigericin and monensin)^11^. However, these reagents are broadly membrane permeable and likely simultaneously target multiple acidic organelles (for example, Golgi apparatus with a pH of ∼6.5)^1^, presenting significant challenges for discrete analysis of endosome and lysosome/autophagolysosome biogenesis.

In this study, we report a nanotechnology-enabled strategy for operator-controlled real-time imaging and perturbation of the maturation process of endocytic organelles; and application to investigation of the integration of endosomal maturation with cell signalling and metabolism. Previously, we developed a series of ultra-pH-sensitive (UPS) nanoparticles that fluoresce upon contact with a very narrow pH range (<0.25 pH units)^12^,^13^. These nanoparticles are 30–60 nm in diameter and enter cells exclusively through endocytosis. In this study, we report for the first time that these UPS nanoparticles can clamp the luminal pH at any operator-determined pH (4.0–7.4) based on potent buffering characteristics. We demonstrate application of a finely tunable series of these UPS nanoparticles to quantitative analysis of the contribution of endosomal pH transitions to endosome maturation, nutrient adaptation and growth homeostasis.

## Results

### A nanoparticle library with sharp buffer capacity

**Figure 1:**
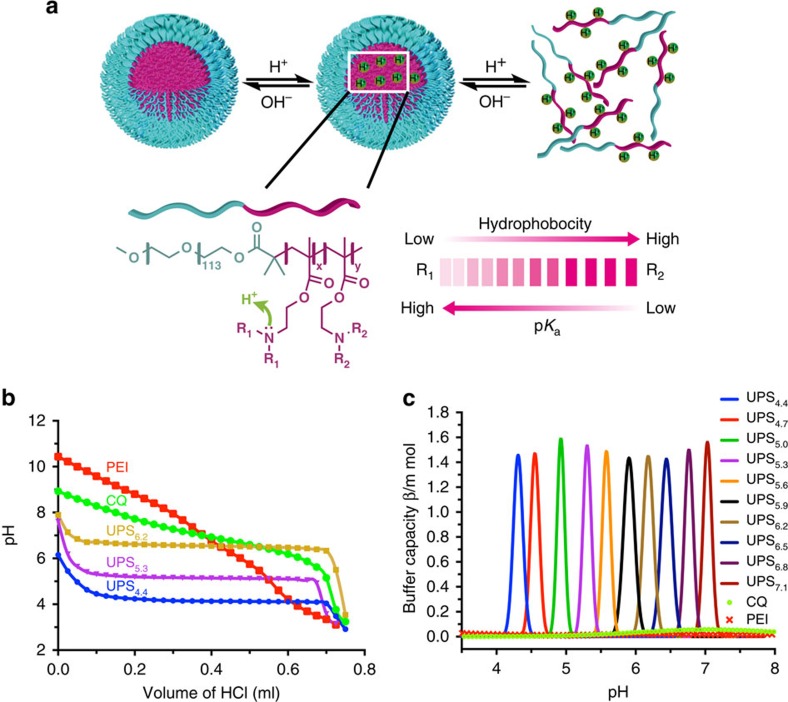
A UPS nanoparticle library with sharply defined buffer capacity across a broad physiological pH range. (**a**) Schematic illustration of the buffer effect of UPS nanoparticles and the chemical structures of PEO-*b*-P(R_1_-*r*-R_2_) copolymers with finely tunable hydrophobicity and pK_a_. The composition for each copolymer is shown in Supplementary Table 1. (**b**) pH titration of solutions containing UPS_6.2_, UPS_5.3_ and UPS_4.4_ nanoparticles using 0.4 M HCl. The maximum buffer pH corresponds to the apparent pK_a_ of each copolymer. Chloroquine (CQ, pK_a_=8.3 and 10.4), a small molecular base, and polyethyleneimines (PEI) were included for comparison. (**c**) Buffer capacity (*β*) for each component of the UPS library was plotted as a function of pH in the pH range of 4.0–7.4. At different pH values, UPS nanoparticles were 30- to 300-fold higher in buffer strength over CQ. L.E. and E.E. are abbreviations for late endosomes and early endosomes, respectively.

We synthesized a series of amphiphilic block copolymers PEO-*b*-P(R_1_-*r*-R_2_), where PEO is poly(ethylene oxide) and P(R_1_-*r*-R_2_) is an ionizable random copolymer block (Fig. 1a and Supplementary Fig. 1). The molecular composition of each copolymer is shown in Supplementary Table 1. At high pH (for example, 7.4 in phosphate-buffered saline (PBS)), these copolymers self-assemble into core-shell micelle structures (diameter 30–60 nm, surface electrostatic potential −2 to 0 mV, Supplementary Table 1 and Supplementary Fig. 2). At pH below the apparent pK_a_ of each copolymer, micelles dissociate into unimers because of the protonation of tertiary amines. Our previous studies exploited the sharp pH-dependent micelle transitions for the development of a series of tunable, UPS fluorescence sensors^14^.

Here we report the UPS nanoparticles have potent pH-tunable buffer capacity at a narrow pH interval across a broad range of pH (4.0–7.4). Figure 1b shows the pH titration curves of three exemplary UPS_4.4_, UPS_5.3_ and UPS_6.2_ nanoparticles (each subscript indicates the pK_a_ of the corresponding copolymer, Supplementary Table 1) in the presence of 150 mM NaCl. UPS_4.4_, UPS_5.3_ and UPS_6.2_ (2 mg ml^−1^) buffered the pH at their apparent pK_a_ at 4.4, 5.3 and 6.2, respectively, when HCl (0.4 M) was added into the polymer solution. In contrast, CQ, a widely used small molecular base in biological studies, showed a broad pH response in the range of pH 6.0–9.0 (pK_a_=8.3). Moreover, polyethylenimine (PEI), widely used for nucleic acid delivery, also behaved as a broad pH buffer^15^. Determination of buffer capacity (*β*=−dn_H_^+^/dpH, where dn_H_^+^ is the quantity of added H^+^ and dpH is the associated pH change) from the pH titration curves (Fig. 1c and Supplementary Fig. 3) showed exceptionally strong and selective buffering at specific pHs in the range of 4.0–7.4. In particular, the maximal *β* values for UPS_4.4_, UPS_5.6_ and UPS_7.1_ nanoparticles were 1.4, 1.5 and 1.6 mmol HCl per 40 mg of nanoparticle, which are 339-, 75- and 30-fold higher than CQ at pH 4.4, 5.6 and 7.1, respectively (Fig. 1c). To examine the consequences of the UPS nanoparticles on endo/lysosomal membrane and plasma membrane integrity, we employed recombinant cytochrome *C* release studies^16^ and haemolysis assays^17^. No detectable perturbation of endosomal or plasma membrane lysis, at 200 or 400 μg ml^−1^ of UPS nanoparticles, was detected as compared with positive or negative controls (Supplementary Fig. 4, see Supplementary Methods). This collection of UPS nanoparticles thus provides a unique set of pH-specific ‘proton sponges' for the functional range of organelle pH from early endosomes (E.E., 6.0–6.5)^18^ to late endosomes (L.E., 5.0–5.5)^18^ to lysosomes (4.0–4.5)^9^.

### pH buffering of endocytic organelles

For simultaneous imaging and buffering studies, we established a new nanoparticle design with a dual fluorescence reporter: an ‘always-ON' reporter to track intracellular nanoparticle distribution regardless of the pH environment and a pH-activatable reporter (OFF at extracellular medium pH 7.4 and ON at specific organelle pH post endocytosis, see Supplementary Methods). Our initial attempts at conjugating a dye (for example, Cy3.5) on the terminal end of PEO produced an ‘always-ON' signal, however, the resulting nanoparticles were unstable because of dye interactions with serum proteins (data not shown). To overcome this limitation, we employed a heteroFRET design using a pair of fluorophores that were introduced in the core of micelles. As an example, we separately conjugated a FRET pair (for example, BODIPY and Cy3.5 as donor and acceptor, respectively) to the P(R_1_-*r*-R_2_) segment of the UPS_6.2_ copolymer. Mixing of the two dye-conjugated copolymers (optimal molar ratio of donor/acceptor=2:1) within the same micelle core allowed the heteroFRET-induced fluorescence quenching of donor dye (that is, BODIPY) in the micelle state (pH>pK_a_), but fluorescence recovery in the unimer state after micelle disassembly at lower pH (Supplementary Fig. 5a upper panel). To generate the ‘always-ON' signal, a low weight fraction of Cy3.5-conjugated copolymer in the micelles was used (for example, 40%) to avoid homoFRET-induced fluorescence quenching for the acceptor dye in the micelle state^13^ (Supplementary Fig. 5b). The resulting UPS nanoparticle showed constant fluorescence intensity in the Cy3.5 channel across a broad pH range, whereas achieving UPS activation at specific pH for the BODIPY signal (Supplementary Fig. 5c).

**Figure 2:**
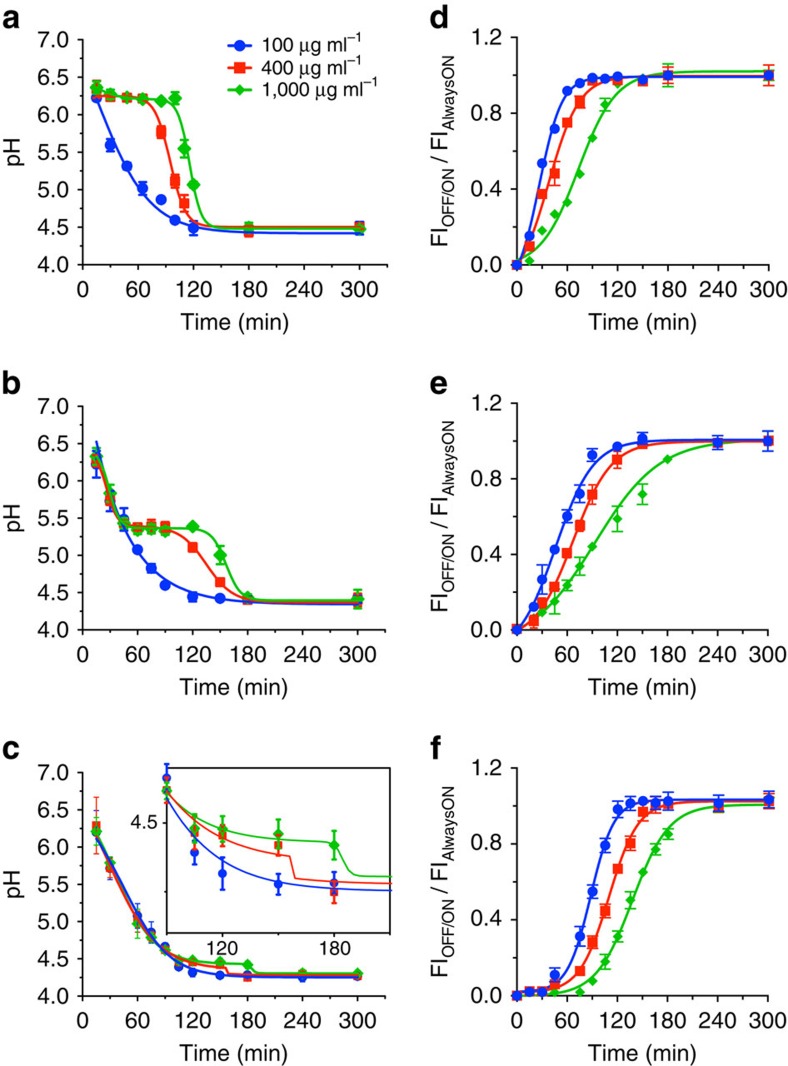
pH-sensitive buffering of endocytic organelles in HeLa cells. Real-time measurement of endo/lysosomal pH in HeLa cells treated with the indicated doses of UPS_6.2_ (**a**), UPS_5.3_ (**b**) and UPS_4.4_ (**c**). The inset is a zoomed-in view of the curve from pH 4.1 to 4.7 and 90 to 210 min in **c**. Lysosensor ratiometric imaging probe was used for *in situ* pH measurement. Quantitative analyses of the activation kinetics of always-ON/OFF-ON UPS_6.2_ (**d**), UPS_5.3_ (**e**) and UPS_4.4_ (**f**). The fluorescent intensity of punctae in BODIPY channel (OFF-ON) was normalized to that of Cy3.5 (always-ON). The error bars represent s.d. from 50 organelles at each time point. In **a** and **b**, the 100 μg ml^−1^ curves are significantly different from the 400 and 1,000 μg ml^−1^ ones, with a *P*-value <0.0001, whereas in **c**, the 100 μg ml^−1^ curve is significantly different from the 400 and 1,000 μg ml^−1^ ones at 120 and 180 min time points, with a *P*-value <0.05. Two-way analysis of variance and Dunnett's multiple comparison tests were performed to assess the statistical significance. Blue, red and green plots indicate 100, 400 and 1,000 μg ml^−1^ of UPS nanoparticles in all panels.

UPS_6.2_, UPS_5.3_ and UPS_4.4_ were chosen for cellular imaging and buffering studies as their apparent pK_a_'s correspond to early endosomes, late endosomes and lysosomes, respectively^18^. All cell-based experiments were performed in the presence of both HEPES (25 mM) and sodium bicarbonate buffers in a 5% CO_2_-controlled environment. HeLa cells were incubated with an increasing dose (100, 400 and 1,000 μg ml^−1^) of UPS_6.2_, UPS_5.3_ or UPS_4.4_ for 5 min at 37 °C to allow particle uptake via endocytosis^19^, then washed with fresh medium (10% FBS in DMEM). At 100 μg ml^−1^, we observed half maximal UPS_6.2_ activation (BODIPY channel) by 30 min, half maximal UPS_5.3_ activation by 60 min and half maximal UPS_4.4_ activation by 90 min (Fig. 2d–f and Supplementary Fig. 6). In contrast, at 1,000 μg ml^−1^, activation of BODIPY signal was delayed by at least 60 min despite clear indication of particle uptake in the HeLa cells by the Cy3.5 signal (Supplementary Fig. 6). *In situ* quantification of the endosomal pH with Lysosensor showed dose-dependent sustained pH plateaus at pH 6.2, 5.3 and 4.4 upon exposure of cells to 400 and 1,000 μg ml^−1^ of UPS_6.2_, UPS_5.3_ and UPS_4.4_ (Fig. 2a–c, Supplementary Fig. 7 and Supplementary Table 2), respectively. For either nanoparticle, 100 μg ml^−1^ was insufficient to delay organelle acidification.

To further quantify the acidification rates, we measured the number of micelle nanoparticles per HeLa cell based on the fluorescence intensity of internalized UPS divided by the cell number (see Supplementary Methods for detials). Particle accumulation appropriately corresponded to the incubation dose (Supplementary Table 2). Based on the number of amino groups per micelle (64,000)^20^ and an average of 200 endosomes/lysosomes per cell^21^, we measured the acidification rate as ∼140–190 protons per second for each organelle. To our best knowledge, this is the first example of quantitative measurement of proton accumulation rates in endocytic organelles based on the endosome specificity and unique ‘buffer and report' design of the UPS nanoparticles. This result is consistent with extrapolations (280–300 protons per second) based on 2 protons per ATP hydrolysed per v-ATPase^22^, 3 ATP molecules consumed per rotation^23^, 2.4 revolutions per second^24^ and an average of 20 v-ATPases per organelle^25^.

### pH thresholds exist in nutrient-induced mTORC1 activation

**Figure 3:**
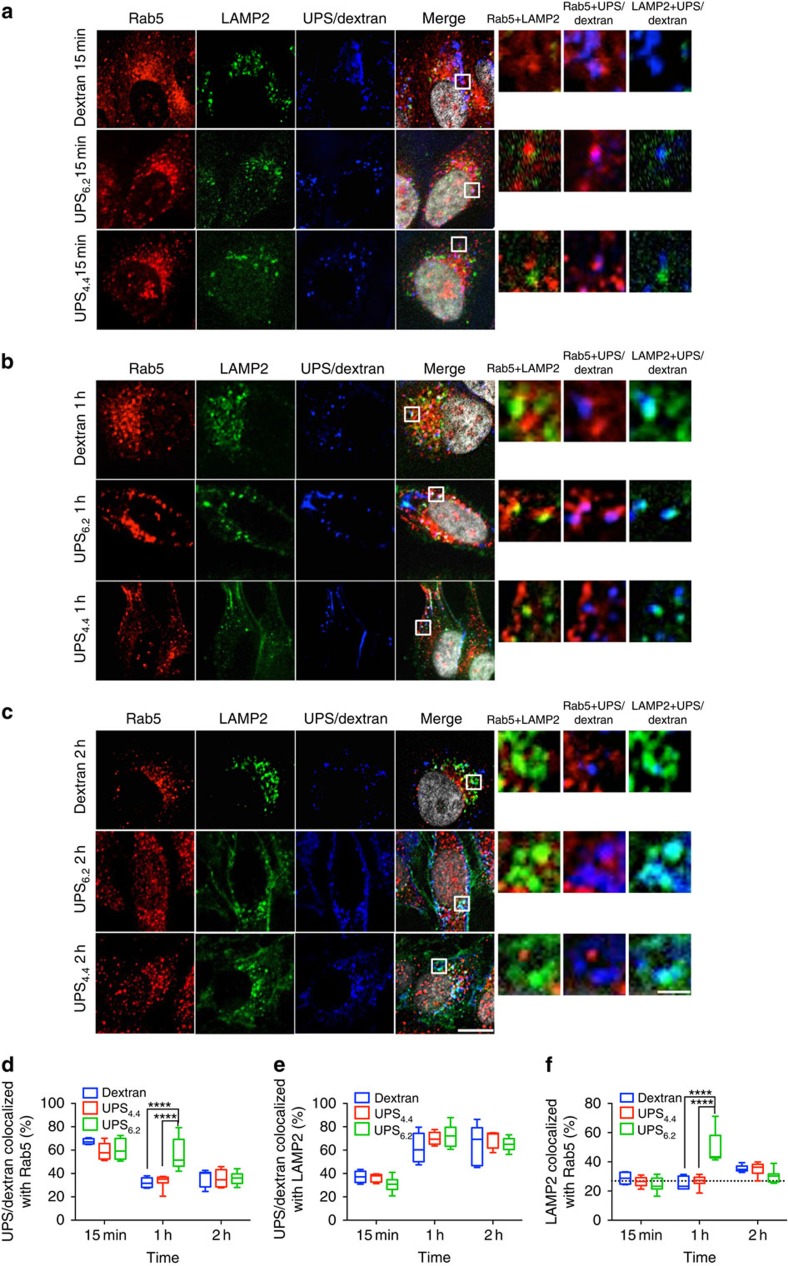
Buffering the pH of endocytic organelles affects their membrane protein dynamics. HeLa cells were treated with 500 μg ml^−1^ dextran-TMR or 1,000 μg ml^−1^ UPS_6.2_-Cy5 or UPS_4.4_-Cy5 for 5 min for cell uptake. Then they were fixed after 15 min (**a**), 1 h (**b**) and 2 h (**c**). Immunofluorescence (IF) images show the localization of UPS nanoparticles in early endosomes (Rab5) or lysosomes (LAMP2). Scale bar, 10 and 5 μm (inset). Imaris software was used to analyse co-localization of z-stacked confocal images. The fraction of UPS/dextran co-localized with Rab5 (**d**) and LAMP2 (**e**) and the fraction of Rab5 co-localized with LAMP2 (**f**) were calculated from thresholded Mander's coefficient (see Supplementary Methods), *n*=10, *α*=0.05, ****P<0.0001. Two-way analysis of variance and Sidak's multiple comparison tests were performed to assess the statistical significance. The dashed line in (**f**) represents the basal level of Rab 5 and LAMP2 co-localization in HeLa cells without any treatment.

We examined the consequences of UPS buffering of luminal pH on endosome protein coat maturation and endo/lysosome-dependent signal transduction. For this purpose, we selected UPS nanoparticles that discretely report and buffer at pH 6.2, 5.3, 5.0, 4.7 and 4.4. This range covers established luminal pH values in early endosomes, late endosomes and lysosomes. A discriminating feature of early endosome biogenesis is recruitment of the Rab5 GTPase^26^, which corresponds to a luminal pH range of 6.0–-6.5 (ref. 18). Fully mature lysosomes are LAMP2 positive with a luminal pH range of 4.0–4.5 (ref. 9). To enable quantification of co-localization of UPS-positive endosomes with endosomal maturation markers, UPS_6.2_–Cy5 and UPS_4.4_–Cy5 were developed with a low dye/polymer ratio that allowed for detectable fluorescence in the micelle state^20^ (Fig. 3a–c). We used fluorescent dextran as a temporally synchronized comparator that does not perturb luminal pH. Within 15 min at a concentration of 1,000 μg ml^−1^, over 60% of UPS_6.2_-, UPS_4.4_- and dextran-positive endosomes were also Rab5 positive (Fig. 3a,d). UPS_4.4_- and dextran-positive endosomes further transitioned to a Rab5-negative/LAMP2-positive maturation state within 60 min (Fig. 3b,e–f). Notably, UPS_6.2_-positive endosomes also became LAMP2 positive in a similar timeframe despite inhibition of the luminal acidification that normally accompanies this transition (Fig. 3b,d). However, UPS_6.2_ delayed release of Rab5, resulting in transient accumulation of anomalous Rab5/LAMP2-positive endosomes at 60 min (Fig. 3b,f and Supplementary Fig. 8). These observations indicate the presence of a regulatory mechanism that recruits LAMP2 to nascent endolysomes independent of the luminal pH and the presence of a luminal pH-sensitive Rab5 release mechanism.

To evaluate integration of endosome maturation with cell regulatory systems, we examined signal transduction events coupled to endosomal compartments. The epidermal growth factor receptor, EGFR, has been shown to activate mitogenic signalling cascades following its ligand-dependent internalization in early endosomes^27^. As might be expected from a pH-regulated system, the delayed endosomal maturation induced by UPS_6.2_ also resulted in delayed EGFR degradation and prolonged activation of ERK1/2 and AKT in response to EGF (Supplementary Fig. 9b).

To further examine the consequence of luminal pH clamping on endo/lysosome biology, we investigated a key regulatory system recently reported to be linked to lysosome biogenesis—namely, nutrient-dependent activation of cell growth via mammalian target of rapamycin complex 1 (mTORC1). In mammalian cells, mTORC1 localizes to endo/lysosomal membranes in response to internalized free amino acids^28^. Furthermore, the physical interactions between the v-ATPase and Rag GTPases on endo/lysosomal membranes are essential for mTORC1 activation in response to nutrient availability^29^. To evaluate amino-acid-induced mTORC1 activation, we employed two quantitative reporters of mTORC1 pathway activation: phosphorylation/activation of the mTORC1 substrate p70S6 kinase (p70S6K) and nuclear/cytoplasmic distribution of the mTORC1 substrate transcriptional factor EB (TFEB).

**Figure 4:**
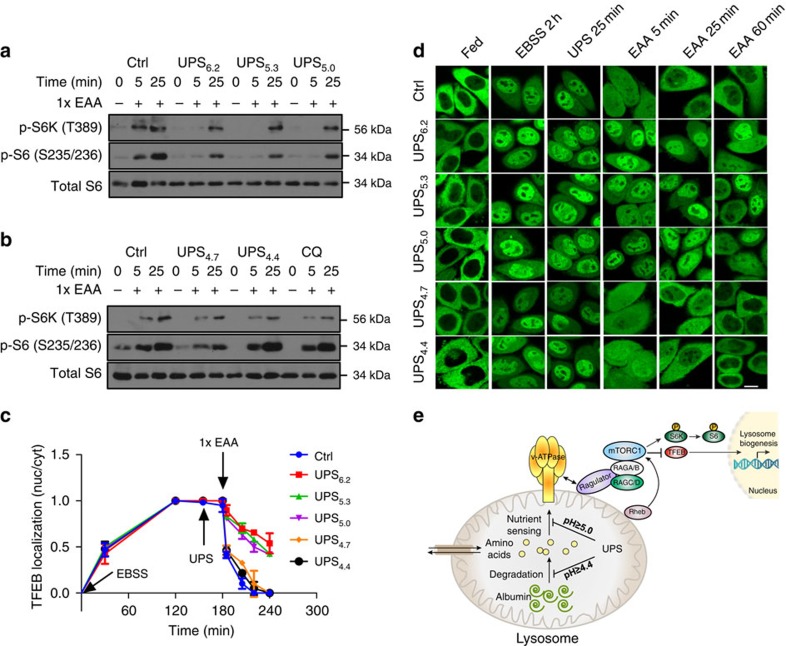
Clamping luminal pH of endo-lysosomes with UPS selectively inhibits amino-acid-dependent mTORC1 activation. HeLa cells were starved in EBSS for 2 h and then stimulated with essential amino acids (EAAs) for indicated time intervals in the presence of (**a**) UPS_6.2_/UPS_5.3_/UPS_5.0_ and (**b**) UPS_4.7_/UPS_4.4_. Water and 50 μM chloroquine (CQ) were used as control. Accumulation of the indicated phosphoproteins was assessed by immunoblot of whole-cell lysates. (**c**) Quantitative analysis of the nuclear/cytosolic distribution of GFP-TFEB following the indicated treatments. Error bars represent s.d., *n*=10. (**d**) Representative images for **c**. Scale bar, 10 μm. (**e**) Working model of pH transitions required for free amino acid versus albumin-derived amino-acid-dependent activation of the mTORC1 signalling pathway.

Incubation of HeLa cells for 2 h in a nutrient-free balanced salt solution (Earle's balanced salt solution (EBSS)) was sufficient to inhibit mTORC1 activity as indicated by reduced accumulation of activation site phosphorylation on both p70S6K and its substrate S6. Addition of essential amino acids was sufficient to induce pathway activation within 5 min (Fig. 4a, Supplementary Figs 10a,b and 12a–c). Pretreatment with 1,000 μg ml^−1^ of UPS_4.7_ or UPS_4.4_ had little to no effect on the mTORC1 response to free amino acids. In contrast, pretreatment with 1,000 μg ml^−1^ UPS_6.2_, UPS_5.3_ and UPS_5.0_ both delayed and significantly suppressed the mTORC1 pathway response to free amino acids (Fig. 4a,b, Supplementary Figs 10a,b and 12). This correlated with inhibition of amino-acid-induced recruitment of mTOR to endo/lysosomes (Supplementary Fig. 10d). The selective UPS inhibition of the mTORC1 pathway response was mirrored by TFEB nuclear/cytoplasm distribution. Phosphorylation of this transcription factor by mTORC1 results in nuclear exclusion, thereby inhibiting the TFEB transcriptional programme in nutrient replete conditions^30^,^31^,^32^. In Hela cells, with stable expression of GFP-tagged TFEB, pretreatment with UPS_6.2_, UPS_5.3_ and UPS_5.0_ inhibited redistribution of TFEB to the cytoplasm upon addition of free amino acids. In contrast, in cells pretreated with UPS_4.7_ and UPS_4.4_, TFEB redistribution proceeded normally (Fig. 4c,d).

The above data suggest acidification of endosomes below a threshold of pH 5 is necessary for free amino-acid-induced activation of mTORC1. We performed similar experiments employing bovine serum albumin (BSA) as a macromolecular nutrient source rather than free amino acids. Similar to free amino acids, BSA exposure was sufficient to reactivate mTORC1 following nutrient starvation (Supplementary Fig. 11). However, in contrast to free amino acids, UPS_4.4_ delayed mTORC1 activation in response to BSA (Supplementary Fig. 11a,d). Given that cells treated with UPS_4.4_ responded normally to free amino acids, we surmised the delayed response to BSA is the consequence of inhibition of the proteolysis of BSA by acid hydrolases in the lysosome. Consistent with this, we found significant inhibition of cathepsin B activity in the presence of UPS_4.4_ (Supplementary Fig. 10c) as well as inhibition of autophagic degradation of p62/SQSTM1 otherwise induced by serum-deprivation (Supplementary Fig. 9a). Together, these observations indicate that distinct lysosomal pH thresholds are required for acid hydrolase activity versus free amino-acid sensing (Fig. 4e).

### Clamping lysosomal pH modulates cellular metabolite pools

**Figure 5:**
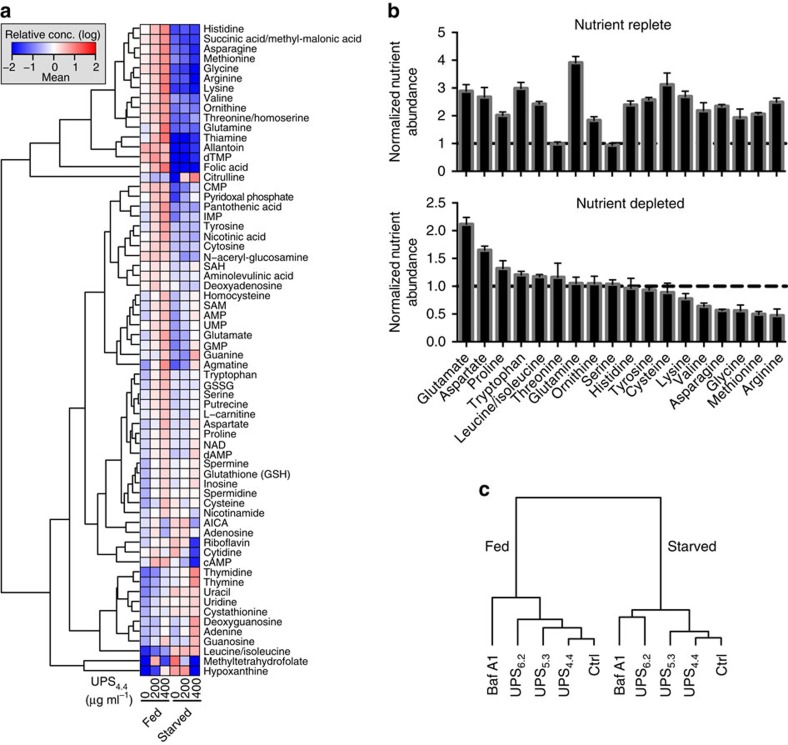
Selective buffering of lysosomal pH modulates the cellular metabolite pool. (**a**) Dendrogram indicates relative abundance of the indicated metabolites in nutrient replete (fed) or deprived (starved) medium as normalized to the total protein content. Cells were treated with UPS_4.4_ at the indicated doses. (**b**) Normalized abundance of the selected amino acids under nutrient replete and nutrient-deprived conditions. Error bars represent s.d., *n*=6. (**c**) An unsupervised hierarchical clustering of different treatments including 1,000 μg ml^−1^ UPS_6.2_, UPS_5.3_ and UPS_4.4_ or 100 nM baf A1 under fed and starved conditions. Dentrogram was generated based the abundance of 108 metabolites.

Lysosomes recycle intracellular macromolecules and debris to produce metabolic intermediates deployed for energy production or for construction of new cellular components in response to the nutrient status of the cellular environment^3^. Abnormal accumulation of large molecules, including lipids and glycoproteins, in lysosomes are associated with metabolic disorders. To broadly assess alterations associated with highly selective perturbation of lysosomal acidification, we quantified accumulation of small metabolites in cells loaded with UPS_4.4_ under nutrient-starved versus nutrient-replete growth conditions (see Supplementary Methods for details). Following a 12-h exposure to 0, 200 and 400 μg ml^−1^ of UPS_4.4_, HeLa cells were lysed and intracellular metabolites were quantified using liquid chromatography-triple quadrupole mass spectrometry (LC/MS/MS). Sixty-eight metabolites were quantifiable from 3 × 10^6^ HeLa cells, revealing a number of dose-dependent and nutrient-dependent consequences of pH arrest at 4.4 in lysosomes (Fig. 5a). Under nutrient replete conditions, as the dose of UPS_4.4_ increased, the relative abundance of most metabolites also increased when normalized to cellular protein content. This included most amino acids (Fig. 5b upper panel), consistent with an inhibition of the anabolic signals required to use them for protein synthesis and/or defects in lysosomal export of amino acids. In nutrient-deprived conditions, UPS_4.4_ enhanced the relative abundance of nucleotides and their precursors (for example, bottom cluster in Fig. 5a) and massively suppressed the second messenger cAMP. The loss of many essential amino acids including lysine, valine, methionine and arginine was also observed, consistent with the inhibition of starvation-induced catabolism of macromolecules like albumin (Fig. 5b lower panel). These results confirm mechanistic connections between organelle acidification and metabolite pools, and fortify the hypothesis that proper lysosomal acidity is required for homeostasis of numerous metabolic pathways, either in the presence or in the absence of nutrients. We performed extensive additional metabolic profiling with or without exposure of cell cultures to 1,000 μg ml^−1^ UPS_6.2_, UPS_5.3_ UPS_4.4_ and 100 nM bafilomycin A1 (baf A1) under both nutrient-deprived and nutrient-replete conditions. Using the quantitative metabolite profiles as response vectors, we found that the UPS probes clustered nicely according to expectations associated with their pH clamping activity (Fig. 5c). UPS_6.2_, UPS_5.3_ and UPS_4.4_ probes induced concordant drops in key amino acids. Baf A1 clustered with UPS_6.2_, consistent with these agents maximally perturbing endosomal acidification (Supplementary Fig. 13). Notably, baf A1 also induced distinct metabolic changes as compared with all other probes and carrier controls (Fig. 5c and Supplementary Fig. 13), which may reflect its broader ‘target space' in cells.

### Vulnerability of KRAS*^mut^*/LKB1*^mut^* NSCLC cells to pH arrest

**Figure 6:**
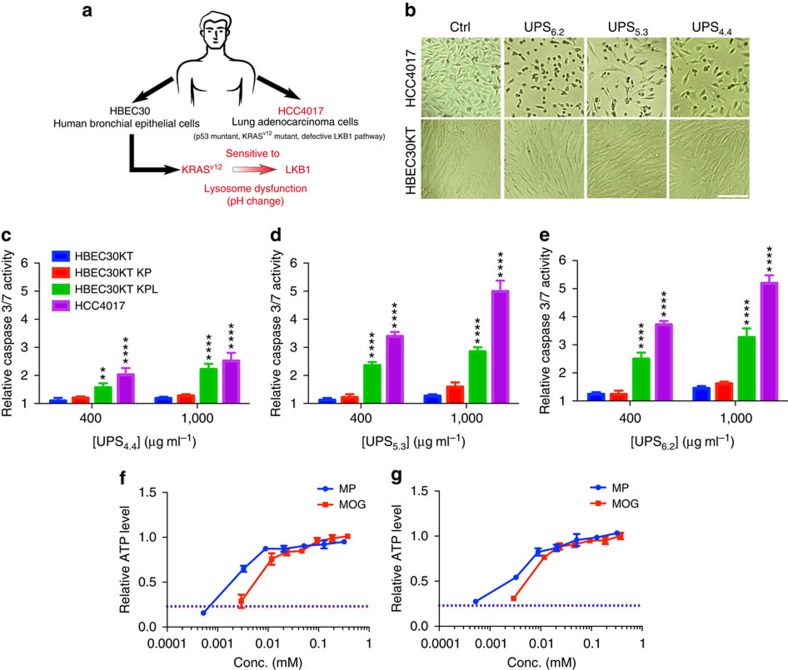
UPS nanoparticles selectively kill NSCLC cells that are sensitive to lysosomal stress. (**a**) Schematic of the cell models employed and their corresponding vulnerabilities to lysosomal maturation. (**b**) Bright field images indicating the relative viability of HBEC30 KT and HCC4017 cells with and without exposure to UPS at effective doses (UPS_6.2_ and UPS_5.3_=400 μg ml^−1^, UPS_4.4_=1,000 μg ml^−1^). Scale bar, 100 μm. (**c**–**e**) Caspase3/7 activity in HBEC30KT, HBEC30KT KP, HBEC30KT KPL and HCC4017 cells was measured 72 h after exposure to the indicated doses of UPS. Two-way analysis of variance and Sidak's multiple comparison tests were performed to assess statistical significance of observed differences between HBEC30KT and HCC4017, and HBEC30KT KP and HBEC30KT KPL, *α*=0.05, **P<0.01, ****P<0.0001. (**f**,**g**) Cellular ATP levels were measured after exposure of HCC4017 (**f**) and HBEC30 KT KPL (**g**) to 1,000 μg ml^−1^ UPS_6.2_ for 72 h together with the indicated concentrations of methyl pyruvate (MP), dimethyl-2-oxoglutarate (MOG) or water (dash line). Values were normalized to no treatment (that is, without UPS) controls. Error bars indicate s.d., *n*=4.

We recently described a selective metabolic vulnerability in non-small-cell lung cancer (NSCLC) cells, whereby co-occurring mutations in the KRAS oncogene and LKB1 tumour-suppressor result in cellular addiction to lysosomal catabolism for maintenance of mitochondrial health^33^. Genetic or chemical inhibition of v-ATPase activity was sufficient to selectively induce programmed cell death in this oncogenic background. This was proposed to be a direct consequence of inhibition of a lysosome-dependent supply of trichloroacetic acid cycle substrates for ATP production. The UPS library afforded an opportunity to directly test this hypothesis in the absence of confounders associated with the pleiotropic contributions of v-ATPases to cytosolic pH and mTORC1/AMPK protein complexes in cancer cells^29^,^34^. As a model system, we employed normal (HBEC30KT) and tumour-derived (HCC4017) cell lines from the same patient together with an isogenic progression series in which the KRAS and LKB1 lesions were artificially introduced into the normal cell background (Fig. 6a)^35^. A comparison of cell number and morphology between HCC4017 and HBEC30KT treated with UPS_6.2_, UPS_5.3_ and UPS_4.4_ at high dose revealed highly selective toxicity of these UPS nanoparticles to HCC4017 (Fig. 6b). The expression of oncogenic KRAS together with inhibition of LKB1 was sufficient to induce sensitivity of bronchial epithelial cells to UPS-induced programmed cell death (Fig. 6c–e). Importantly, this phenotype was rescued in both the tumour-derived cells (Fig. 6f) and the genetically engineered cells (Fig. 6g) upon addition of cell permeable analogues of trichloroacetic acid cycle substrates (methyl pyruvate and α-ketoglutarate). Although UPS nanoparticles are able to clamp endo/lysosomal pH, they did not have detectable effects on cytosolic pH (Supplementary Fig. 14). Thus, selective vulnerability of KRAS/LKB1 co-mutant NSCLC cells to lysosomal function likely arises from addiction to catabolism of extracellular macromolecules.

## Discussion

Luminal acidification is a hallmark of maturation of endocytic organelles in mammalian cells with pH-selective mechanistic consequences on receptor recycling, organelle trafficking and protein/lipid catabolism^1^,^2^. Existing tools and reagents employed to manipulate luminal acidification (for example, CQ, NH_4_Cl, baf A1) are membrane permeable and perturb a broad range of pH-dependent cellular activities. Consequently, investigation of endosome/lysosome biology using these agents can suffer from compounded, non-specific effects on multiple acidic organelles (for example, Golgi). In contrast, UPS nanoparticles enter cells exclusively through endocytosis and allow robust and fine-scale buffering of luminal pH at operator-determined thresholds along the endocytic pathway without disrupting cell or organelle membranes. The exceptional potency and specificity of the UPS nanoparticle buffering characteristics, together with previously reported UPS fluorescence response^12^,^13^, holds considerable advantage over reported pH-sensitive probes (for example, small molecular dyes^36^, peptides^37^,^38^ or photoelectron transfer nanoprobes^39^,^40^ with ten-fold signal change over 2 pH unit). The ultra-pH responsive property of the UPS system is a unique nanoscale phenomenon for self-assembled systems. The hydrophobic micellization (phase transition) dramatically sharpens the pH transition leading to cooperative protonation of tertiary amines. As a result, the UPS nanoparticles yielded a high-resolution buffer effect within 0.3 pH unit. In contrast to small molecular pH buffers/sensors that are mostly controlled by electron withdrawing/donating substituents^36^, the buffered pH range (centred around apparent pK_a_) of the UPS platform can be fine-tuned by the hydrophobicity of the PR segment. This chemical simplicity and versatility to achieve fine-tunability by the UPS design is advantageous over reported pH-sensitive non-covalent polymer systems (for example, polyaminoacids^41^ such as poly(L-histidines)^42^,^43^) or covalent polymer systems (for example, pH-labile bonds such as trimethoxy benzylidenes)^44^,^45^. The unique pH-specific, tunable ‘proton sponge' effect is also distinct from other low-resolution polybase buffers (for example, PEI, Fig. 1c). To enhance simultaneous imaging and buffering capability, we constructed an always-ON/OFF-ON dual reporter design employing a heteroFRET strategy.

Detailed evaluation of the UPS library in cells illuminated mechanistic integration of dynamic luminal pH transitions in endosomes with multiple cell physiological processes. For example, the ‘perturb and report' characteristics of the library allowed for time-resolved quantitation of endosome maturation, and uncovered previously unappreciated consequences of luminal pH on endosomal coat protein exchange. Notably, we found that recruitment of the ‘mature' lysosome marker, LAMP2, occurs independently of luminal acidification. On the other hand, release of the early endosome marker Rab5 is delayed by luminal alkalization, resulting in the *de novo* accumulation of Rab5/LAMP2-positive endosomes. This indicates the presence of currently undescribed, but explorable, pH-sensitive and pH-insensitive mechanisms governing endosome/lysosome biogenesis. The ability to fine-tune UPS buffering capacity also allowed discrimination of distinct pH thresholds required for free amino acid versus albumin-dependent activation of mTORC1 pathway. We speculate that acidification to pH 5.0 or below is required to release free amino acids for ‘inside-out' communication with v-ATPase protein complexes, or for induction of conformational changes in v-ATPase during amino-acid sensing^29^. Acidification to pH 4.4 or below is necessary for albumin-dependent activation of mTORC1, most likely due to the need for hydrolase activation and subsequent protein catabolism. Some hydrolases are reported to have pH-sensitive specific activity changes within the ranges spanned by the UPS probes employed here. For example, the *in vitro* activity of cathepsin B shows 10–20% fluctuation from pH 4.0 to 6.0 (ref. 46). We did not observe this range of activity in cells treated with UPS_6.2_, UPS_5.3_ and UPS_4.4_, which may result from the detection limit of the assays we used. The scalability of UPS synthesis enabled broad-spectrum quantification of the cellular metabolite milieu upon inhibition of lysosomal consumption of extracellular macromolecules. The exclusive uptake of UPS within endocytic organelles afforded the opportunity to specifically evaluate the participation of endosomal/lysosomal pH in growth regulatory signalling pathways and cell metabolism.

In summary, we report a new class of biologically compartmentalized, high performance, imageable nanobuffers with high pH precision and resolution at operator-predetermined pH transitions. The combined and controlled perturb and report strategy delivers powerful biophysical tools for quantification of acidification kinetics of endocytic organelles in diverse biological contexts, and for time-resolved perturbation of this kinetics for evaluation of biological relevance. We anticipate these tools will also help generate new insights for biocompatibility and safety assessment of responsive nanomaterials in the rapidly growing fields of nanobiotechnology and drug/gene delivery^47^,^48^.

## Methods

### Chemicals

The Cy5-NHS, BODIPY-NHS and Cy3.5-NHS esters were purchased from Lumiprobe Corp. Monomers 2-(diethylamino) ethyl methacrylate and 2-aminoethyl methacrylate were purchased from Polyscience Company. Monomers 2-(dibutylamino) ethyl methacrylate (DBA-MA)^13^, 2-(dipropylamino) ethyl methacrylate (DPA-MA) and 2-(dipentylamino) ethyl methacrylate^49^ were prepared according to the method described in our previous work, as well as the PEO macroinitiator (MeO-PEO_114_-Br)^13^. *N*,*N*,*N*′,*N*′′,*N*′′′-Pentamethyldiethylenetriamine (PMDETA) was purchased from Sigma-Aldrich. Amicon ultra-15 centrifugal filter tubes (MWCO=100 K) were obtained from Millipore. Other reagents and organic solvents were analytical grade from Sigma-Aldrich or Fisher Scientific Inc.

### Cells, culture medium and biological reagents

The NSCLC cell line HCC4017 and its matched normal bronchial epithelial cell line HBEC30KT were developed from the same patient. The generation of these cell lines and the corresponding HBEC30KT oncogenic progression series was as previously reported^35^. HCC4017 and all HBEC30-derived cell lines were cultured in ACL4 medium (RPMI 1640 with 25 mM HEPES and 2.0 g l^−1^ NaHCO_3_ supplemented with 0.02 mg ml^−1^ insulin, 0.01 mg ml^−1^ transferrin, 25 nM sodium selenite, 50 nM hydrocortisone, 10 mM HEPES, 1 ng ml^−1^ EGF, 0.01 mM ethanolamine, 0.01 mM O-phosphorylethanolamine, 0.1 nM triiodothyronine, 2 mg ml^−1^ BSA, 0.5 mM sodium pyruvate) with 2% fetal bovine serum (FBS, Atlanta Biologicals) and 1% antibiotics (Gibco). HeLa and GFP-TFEB HeLa cells were cultured in DMEM (Invitrogen, containing 25 mM HEPES and 3.7 g l^−1^ NaHCO_3_) with 10% FBS and 1% antibiotics (Invitrogen). EBSS (10 × , Sigma) was diluted to 1 × with Milli-Q water supplemented with 2.2 g l^−1^ sodium bicarbonate (Sigma). All cell-based studies were performed with 25 mM HEPES buffer in a humidified chamber with 5% CO_2_. Antibodies were from Cell Signaling (p70 S6K-pT389 (catalogue# 9205), total p70 S6K (catalogue# 2708), S6-Ribosomal-Protein-pS235/236 (catalogue# 4858), total S6 Ribsomal Protein (catalogue# 2217), Rab5 (catalogue# 3547), mTOR (catalogue# 2983), p62 (catalogue# 8025), EGFR (catalogue# 4267), EGFR-pY1068 (catalogue# 3777), MEK1/2-pS217/221(catalogue# 9154), total MEK1/2 (catalogue# 9126), Akt-p-S473 (catalogue# 4060), pan Akt(catalogue# 4691), GAPDH (catalogue# 5174)) and Abcam (LAMP2 (catalogue# ab13524)). All primary antibodies used for immunoblot are all diluted in 1:1,000. Secondary antibodies were from Jackson ImmunoResearch Laboratories (Peroxidase-conjugated AffiniPure Goat Anti-Rabbit IgG (catalogue# 111-035-144)) and Invitrogen (Alexa Fluor 488 goat anti-rat IgG (catalogue# A11006), Alexa Fluor 594 goat anti-rabbit IgG (catalogue# A11037), Alexa Fluor 635 goat anti-rabbit IgG (catalogue# A31577)). Other biological agents include Hoechst 33342 (Invitrogen), 70 kDa Dextran-TMR (Invitrogen), LysoSensor Yellow/Blue DND 160 (Invitrogen), Magic Red Cathepsin B Assay Kit (Immunochemistry Technology), Bafilomycin A1 (Sigma), Chloroquine (Sigma) Cytochrome C (Sigma) and BCA Protein Assay Kit (Thermo).

### Syntheses of PEO-*b*-(P(R_1_-*r*-R_2_)) block copolymers

The copolymer PEO-*b*-(P(R_1_-*r*-R_2_)) was synthesized using atom transfer radical polymerization method as reported^14^ (Supplementary Fig. 1). The molar fractions of R_1_ and R_2_ were varied to control the hydrophobicity of the PR segment. In a typical procedure using PEO-*b*-P(DPA_60_-*r*-DBA_20_) (UPS_5.9_) as an example, DPA-MA (1.3 g, 6 mmol), DBA-MA (0.48 g, 2 mmol), PMDETA (21 μl, 0.1 mmol) and MeO-PEO_114_-Br (0.5 g, 0.1 mmol) were charged into a polymerization tube. The monomer and initiator were dissolved in a mixture of 2-propanol (2 ml) and dimethylformamide (DMF) (2 ml). Three cycles of freeze–pump–thaw were performed to remove the oxygen, then CuBr (14 mg, 0.1 mmol) was added into the tube protected by nitrogen, and the tube was sealed *in vacuo*. After 8 h polymerization at 40 °C, the reaction mixture was diluted in 10 ml tetrahydrofuran (THF), and the mixture was passed through a neutral Al_2_O_3_ column to remove the catalyst. The organic solvent was removed by rotovap. The residue was dialysed in distilled water and lyophilized to obtain a white powder. The composition and physical properties of UPS library are listed in Supplementary Table 1.

### Titration of UPS nanoparticles

In a typical procedure, 20 ml micelle solution (2 mg ml^−1^) was first prepared at 150 mM concentration of NaCl. Chloroquine (2 mg ml^−1^, 20 ml) or branched PEI (6.2 mg, 20 ml) solutions in 150 mM NaCl were used for comparison. pH titration was carried out by adding small volumes (25 μl increment) of 0.4 M HCl solution under stirring. The pH decrease in the range of 9 to 3 was monitored as a function of added volume of HCl. The pH values were measured using a Mettler Toledo pH metre with a microelectrode. Figure 1b shows the representative titration curves for the UPS nanoparticles. For each sample, the pK_a_ value was calculated as the pH in the middle of the two equivalence points in the titration curve. The pK_a_ values for all the UPS nanoparticles are listed in the Supplementary Table 1. No HCO_3_^−^/H_2_CO_3_ or any other buffers were included in the system to avoid possible interference from external systems to nanoparticle alone. However, all cell-based studies were performed with 25 mM HEPES buffer in a humidified chamber with 5% CO_2_.

### Measurement of endo/lysosomal pH

HeLa cells were plated in 4- or 8-well Nunc Lab-Tek II Chambered Coverglass (Thermo Scientific) and allowed to grow for 48 h. The cells were then loaded with 25 μM LysoSensor Yellow/Blue DND-160 and 100, 400 or 1,000 μg ml^−1^ UPS nanoparticles in serum-free medium at 37 °C for 5 min. The cells were washed twice and immediately imaged. Imaging was performed using an epifluorescent microscope (Deltavision, Applied Precision) equipped with a digital monochrome Coolsnap HQ2 camera (Roper Scientific). Fluorescence images were collected using SoftWoRx v3.4.5 (Universal Imaging). Data were recorded at excitation/emission wavelengths of 360/460 and 360/520 nm. The single band-pass excitation filter for 4,6-diamidino-2-phenylindole (DAPI; 360 nm) is 40 nm, and the band pass of emission filters for DAPI (460 nm) and fluorescein isothiocyanate (520 nm) is 50 and 38 nm, respectively. Cell fluorescence ratios were determined by image analysis using ImageJ software. For each cell, a region of interest was defined as the punctae in cytosol that emitted fluorescent signals from both UPS nanoparticles and LysoSensor. Fluorescent intensity ratio was calculated for each intracellular punctate as *R*=(*F*_1_-*B*_1_)/(*F*_2_-*B*_2_), where *F*_1_ and *F*_2_ are the fluorescence intensities at 360/520 and 360/460, respectively, and *B*_1_ and *B*_2_ are the corresponding background values determined from a region on the same images that was near the punctae in the cytosol. To calibrate the relationship between *R* and pH, we used a modified protocol established by Diwu *et al*^50^. Cells were loaded with LysoSensor and then permeabilized with 10 μM monensin and 10 μM nigericin. These cells were treated for 30 min with the equilibration buffers consisting of 5 mM NaCl, 115 mM KCl, 1.2 mM MgSO_4_ and 25 mM MES (MES buffer) varied between pH 4.0 and 7.4. The cells were kept in the buffer until imaging. The curves for 400 and 1,000 μg ml^−1^ UPS nanoparticles were fit with the bi-dose–response fitting function in OriginLab (v8.0), whereas the curves of 100 μg ml^−1^ were fit with the dose–response function. Two-way analysis of variance and Dunnett's multiple comparison tests were performed to assess the statistical significance using Graphpad Prism (v6.0) software.

### Measurement of intracellular pH

HBEC30 KT and HCC4017 cells were plated in 4- or 8-well Nunc Lab-Tek II Chambered Coverglass (Thermo Scientific) and allowed to grow for 48 h. The cells were then loaded with 1,000 μg ml^−1^ UPS nanoparticles in ACL4 medium at 37 °C with 5% CO_2_ for 24 h. 2′,7′-bis-(2-carboxyethyl)-5-(and-6)-carboxyfluorescein, acetoxymethyl ester (BCECF, AM) stock solution was diluted in EBSS (with 2.2 g l^−1^ NaHCO_3_, without amino acids or buffers containing primary or secondary amines that may cleave the AM esters and prevent loading) to 5 μM. Cells were then loaded with BCECF at 37 °C with 5% CO_2_ for 20 min. Then cells were washed twice and immediately imaged. A spinning-disk confocal microscope was used to acquire images with excitation at 445 and 488 nm, and a 525/40-nm EMCCD emission wheel. Cell fluorescence ratios were determined by using ImageJ software. Fluorescent intensity ratio in the cytosolic region was calculated for each cell as *R*=(*F*_1_-*B*_1_)/(*F*_2_-*B*_2_), where F_1_ and F_2_ are the fluorescence intensities at 488/525 and 445/525, respectively, and *B*_1_ and *B*_2_ are the corresponding background values determined from a region on the same images that was near the cell. To calibrate the relationship between *R* and pH, we used a modified protocol established by Thomas *et al*.^51^ and others^52^,^53^. Different pH buffers (135 mM KCl, 5 mM K_2_HPO_4_, 20 mM HEPES, 1.2 mM CaCl_2_, 0.8 mM MgSO_4_, pH was adjust to 6.0, 6.5, 7.0, 7.5 and 8.0 by adding HCl or KOH) with 10 μM nigericin was used to equilibrate internal and external pH. After 5 min equilibrium, images were taken and analysed as described above to obtain the 488/445 ratio for each calibration pH value. Graphpad Prism (v6.0) software was used to obtain an equation (a sigmoidal plot) that best describes the data.

### Immunofluorescence assays

HeLa cells were plated on glass coverslips in a 12-well tissue culture dish at 500,000 cells per well. After 24 h, the cells were treated with 500 μg ml^−1^ 70 kDa dextran-TMR, 1,000 μg ml^−1^ UPS_6.2_–Cy5 or UPS_4.4_–Cy5 for 5 min in serum-free DMEM, followed by three washes in PBS and subsequent incubation in DMEM+10% FBS for the indicated time. Coverslips were then rinsed with PBS and fixed for 10 min with 4% paraformaldehyde in PBS at room temperature. Following fixation, coverslips were rinsed twice with PBS and cells were permeablized with 0.1% Triton X-100 in PBS for 10 min at 4 °C. After rinsing twice with PBS, the coverslips were incubated with 10% normal goat serum block solution for 45 min at room temperature. The primary antibodies (from different species) were diluted 1:100 in the block solution, and were co-incubated with cells overnight in the dark at 4 °C. Secondary antibodies were diluted 1:200 in the block solution. The cells were then washed with PBS and incubated with secondary antibodies at room temperature for 1 h. The coverslips were washed three times before being mounted on glass slides using Vectashield (Vector Laboratories) and imaged using confocal microscopy.

### Image acquisition and analysis

Confocal laser scanning microscopy was used to investigate the intracellular activation and distribution of UPS nanoparticles. HeLa cells were plated on 4- or 8-well Nunc Lab-Tek II Chambered Coverglass (Thermo Scientific) and allowed to grow for 48 h. Cells were incubated with always-ON/OFF-ON UPS nanoparticles for 5 min in serum-free medium, and washed three times with PBS before imaging. Confocal images in Supplementary Fig. 6 were acquired with a Nikon ECLIPSE TE2000-E confocal microscope with identical settings for each experiment. Data were recorded at excitation wavelengths of 488 nm (BODIPY) and 560 nm (Cy3.5). EZ-C1-free viewer v3.90 (Nikon) and ImageJ software (NIH) were used to convert and analyse the images. The FI_OFF-ON (BODIPY)_/FI_Always-ON (Cy3.5)_ ratio was determined by image analysis using ImageJ (NIH) software. For each cell, a region of interest was defined as the punctae in cytosol that emitted fluorescent signals from both BODIPY and Cy3.5 channels. Fluorescent intensity ratio was calculated for each intracellular punctate as *R*=(*F*_1_-*B*_1_)/(*F*_2_-*B*_2_), where *F*_1_ and *F*_2_ are the fluorescence intensities from BODIPY and Cy3.5 channels, respectively, and *B*_1_ and *B*_2_ are the corresponding background values determined from a region on the same images that was near the punctae in the cytosol. All the ratios of each nanoparticle were normalized to their end-time-point ratio, and the curves were fit with the dose–response function with Graphpad Prism (v6.0) software. Images from the immunofluorescence assay (Fig. 3a–c) were taken by using a spinning disk confocal microscope (Andor). Z-stack images were used after deconvolution in the co-localization analysis. The data were analysed using the Coloc module of Imaris 7.7 (Bitplane). The thresholded Mander's coefficient was used as an indicator of the proportion of the co-localized signal over the total signal^54^,^55^. The distribution and volume analysis was also done in Imaris 7.7. Z-stack images were used after deconvolution, the background was subtracted and a ‘surface' was built for the DAPI, Rab5 and LAMP2 channels, respectively. The volume of each subject in the Rab5 and LAMP2 channels was calculated based on their voxel numbers, and the shortest distance of each to the nucleus surface were calculated using the MATLAB plugin ‘Distance transformation'. The medium distance and volume of Rab5- or LAMP2-positive vesicles in each cell was calculated for each cell. Two-way analysis of variance and Sidak's multiple comparison tests were performed to assess the statistical significance using Graphpad Prism (v6.0) software. A spinning disk confocal microscope (Andor) was used to obtain images in Supplementary Fig. 10d. An average intensity projection was used on the Z-stack images (42 slices) in ImageJ.

### Amino acid or BSA starvation and stimulation of the cells

The method was adapted from Sancak *et al*.^28^. Cells were rinsed and incubated with 1 × EBSS for 2 h. For amino-acid starvation, cells were first pretreated with 10 × glutamine (final concentration 1 × ) for 1 h to facilitate the transport of other amino acids into the cells. UPS nanoparticles were added in the last 25 min of glutamine treatment if needed. Tenfold essential amino-acid solution was added to stimulate cells (final concentration 1 × ). After stimulation, the level of essential amino acids and glutamine in EBSS was the same as in DMEM. The mTOR and LAMP2 assay followed the same protocol here and also the immunofluorescence protocol mentioned above. For BSA starvation, cells were pretreated with UPS nanoparticles for 25 min before adding BSA solution with a final concentration of 2 mg ml^−1^ (ref. 56).

### Apoptosis assay

A Caspase Glo 3/7 assay was used to measure caspase 3/7 activities. A normal bronchiole epithelia-derived (HBEC30KT) cell line, tumour-derived HCC4017, KRAS^*mut*^ HBEC30KT and KRAS^*mut*^/LKB1^*mut*^ HBEC30KT were seeded in 96-well plates (Corning). UPS nanoparticles were added 24 h later. Caspase Glo reagent (Promega) was added after 72 h according to the manufacturer's instructions. Plates were read with PHERAstar FS microplate reader (BMG LABTECH).

## Additional information

**How to cite this article**: Wang, C. *et al*. A nanobuffer reporter library for fine-scale imaging and perturbation of endocytic organelles. *Nat. Commun.* 6:8524 doi: 10.1038/ncomms9524 (2015).

## Supplementary Material

Supplementary Information Supplementary Figures 1-14, Supplementary Tables 1-2, Supplementary Methods and Supplementary References
