# Supplementary material for: A nanobuffer reporter library for fine-scale imaging and perturbation of endocytic organelles
Source: Nat Commun. 2015 Oct 5;6:8524. doi: 10.1038/ncomms9524 (PMC4600749; doi:10.1038/ncomms9524)
Supplement: Supplementary Information — Supplementary Figures 1-14, Supplementary Tables 1-2, Supplementary Methods and Supplementary References [file ncomms9524-s1.pdf]

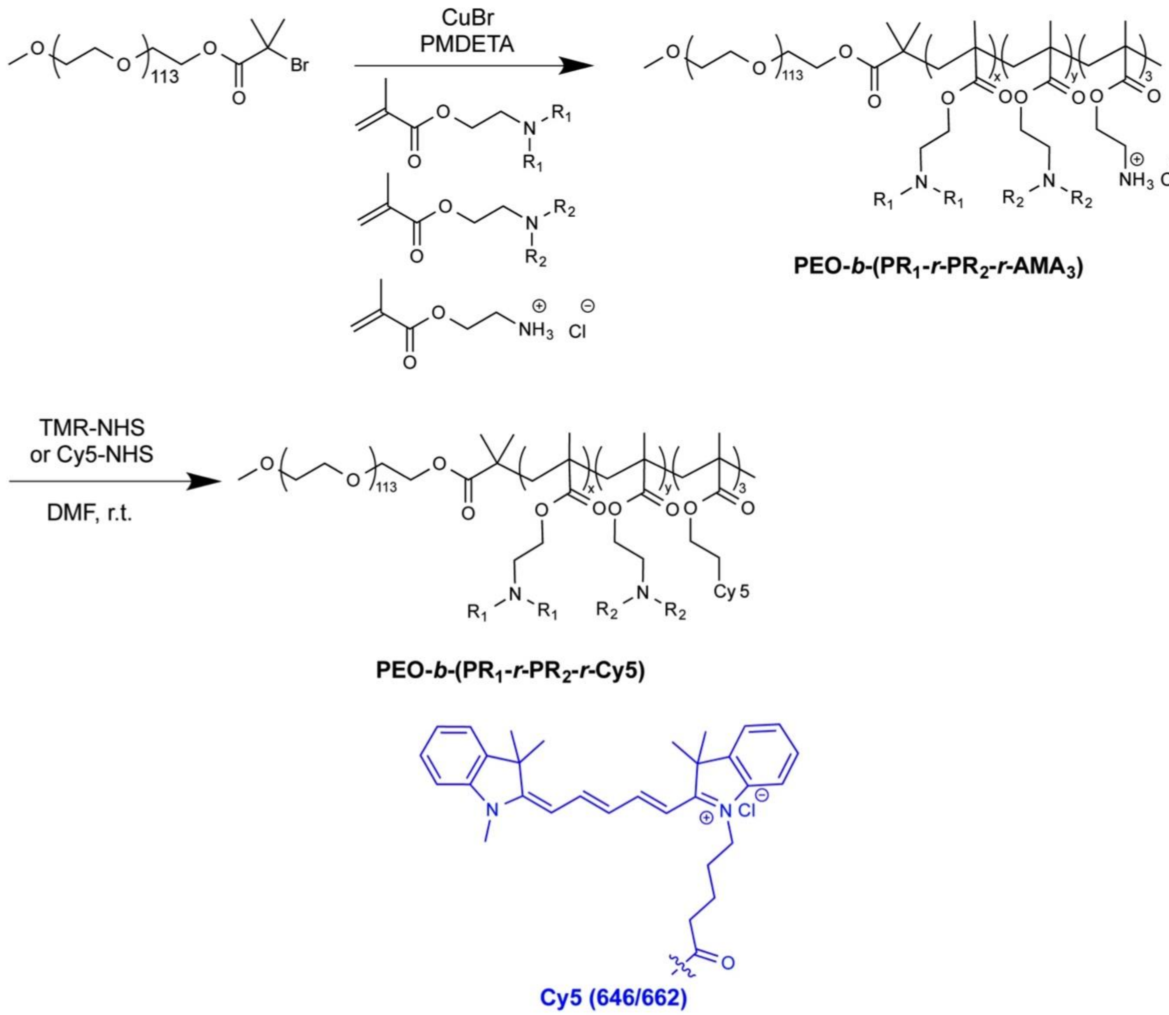

Supplementary Figure 1. Syntheses of dye-conjugated di-block copolymers. The PR segment consists of a random block from two monomers with different molar fractions to fine-tune its hydrophobicity and pH transition (see **Supplementary Table 1**). The structure of Cy5 dye is also shown.

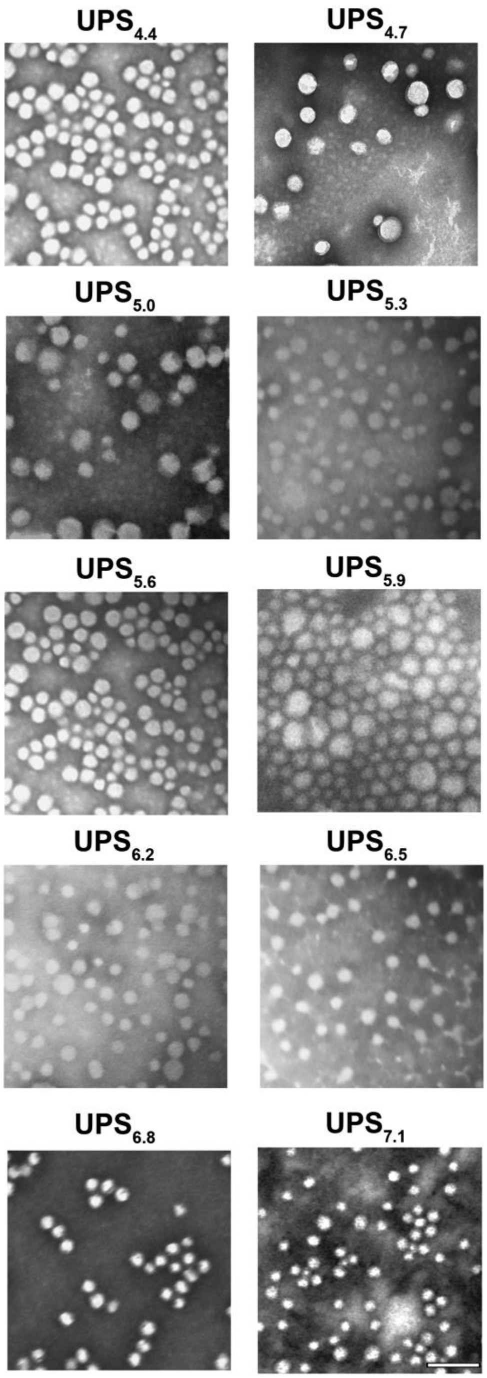

Supplementary Figure 2. Transmission electron microscopy (TEM) images of the UPS nanoparticle library. Nanoparticles were dissolved in PBS buffer (pH 7.4) and dried on a carbon grid prior to TEM analysis. Phosphotungstic acid was used for negative staining. Scale bar = 100 nm for all images.

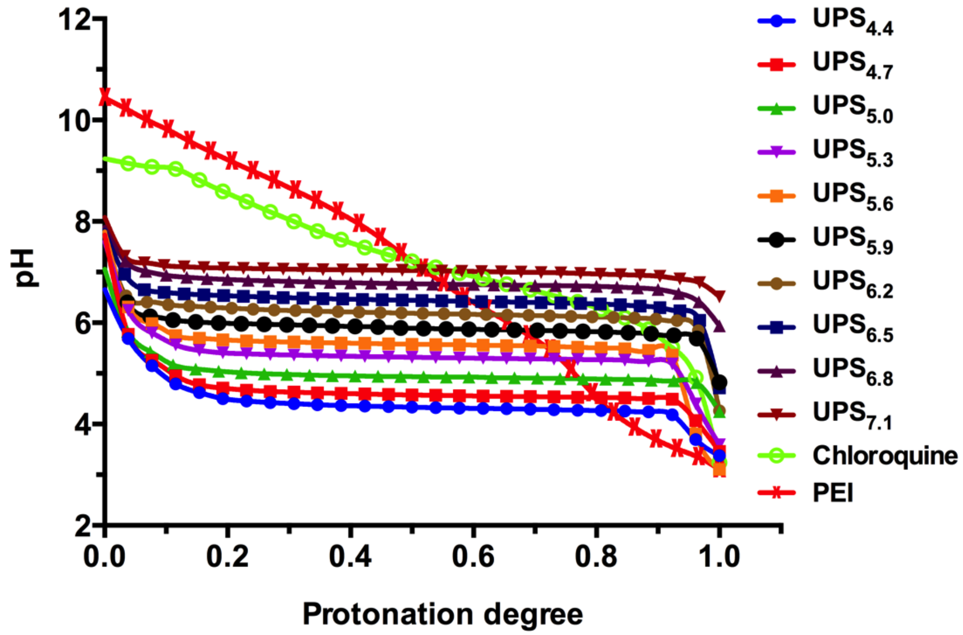

Supplementary Figure 3. pH titration of each component of the UPS nanoparticle library. HCl (0.4 M) was added incrementally to titrate micelle solution (2 mg/mL polymer concentration or 8 mM based on the amount of amine groups) of all ten UPS nanoparticles, choloroquine solution (2 mg/mL or 12.5mM based on the amount of amine groups) and PEI (branched, MW 10,000 Da, Polyscience, Inc.) solution (0.3 mg/mL or 7.3 mM based on the amount of amine groups). A pH/conductivity meter (Mettler Toledo) was used to monitor the change of pH in the solution during titration.

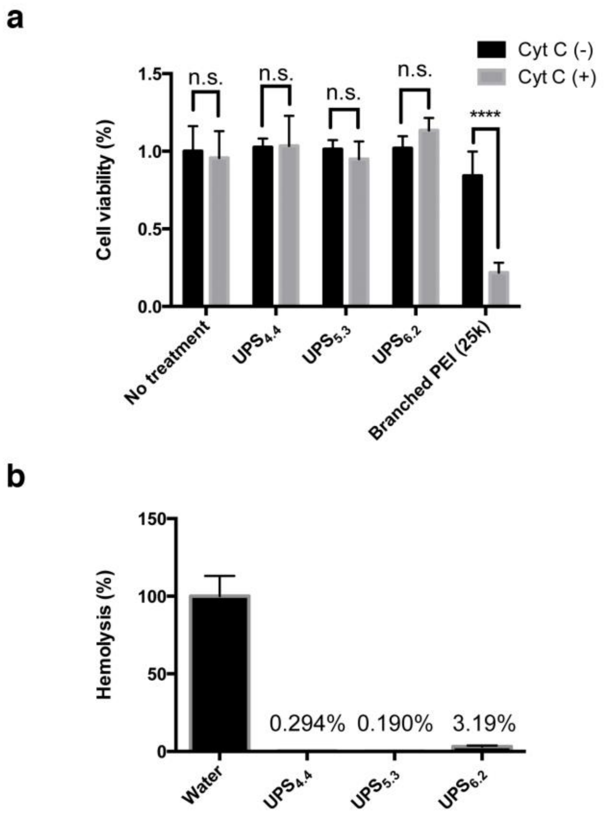

Supplementary Figure 4. Investigation of membrane integrity after incubation with UPS nanoparticles. (**a**) HeLa cells were co-incubated with 200 μg/mL indicated UPS nanoparticles or 32 μg/mL 25 kD branched PEI (Polyethylenimine, as a positive control) and cytochrome C (cyt C) for 5 min. Cell viability was determined after 5 h by using Cell-Titer Glo. (**b**) Freshly-collected red blood cells from mouse blood were diluted in distilled water or 400 μg/mL indicated UPS nanoparticle solutions in phosphate-buffered saline at 1:1 ratio (v/v). Cells were incubated at 37°C for 3 hours, and the supernatant was collected for absorbance measurement on UV/Vis spectrophotometer at 570 nm. Cell lysis in distilled water due to hypotonicity was used as a positive control.

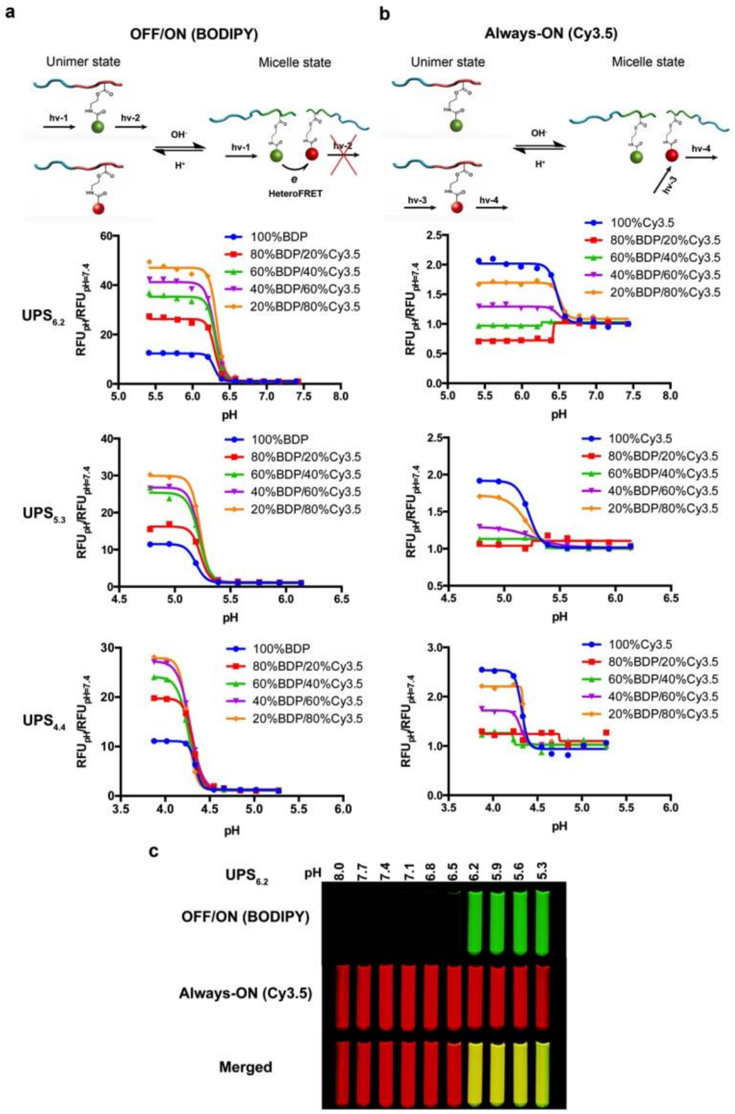

Supplementary Figure 5. Syntheses and characterization of Always-ON/OFF-ON UPS nanoparticles. (**a**) and (**b**) Schematic of the dual-reporter nanoparticle. In the micelle state, the Always-ON dyes serve as the quencher for the ON/OFF fluorophores. When the micelle is disassembled, the Always-ON and ON/OFF fluorophores can fluoresce independently. The ON/OFF ratio of BODIPY (**a**) and Cy3.5 (**b**) varies when the ratio of polymers conjugated with these two dyes varies. Weight fraction of 60% BODIPY-conjugated copolymer with 40% Cy3.5-conjugated copolymer was chosen as the final combination. (**c**) Fluorescence signal amplification of UPS$_{6.2}$ nanoparticles as a function of pH. Images were captured on Maestro *in vivo* imaging system (CRI) using the green and yellow filters.

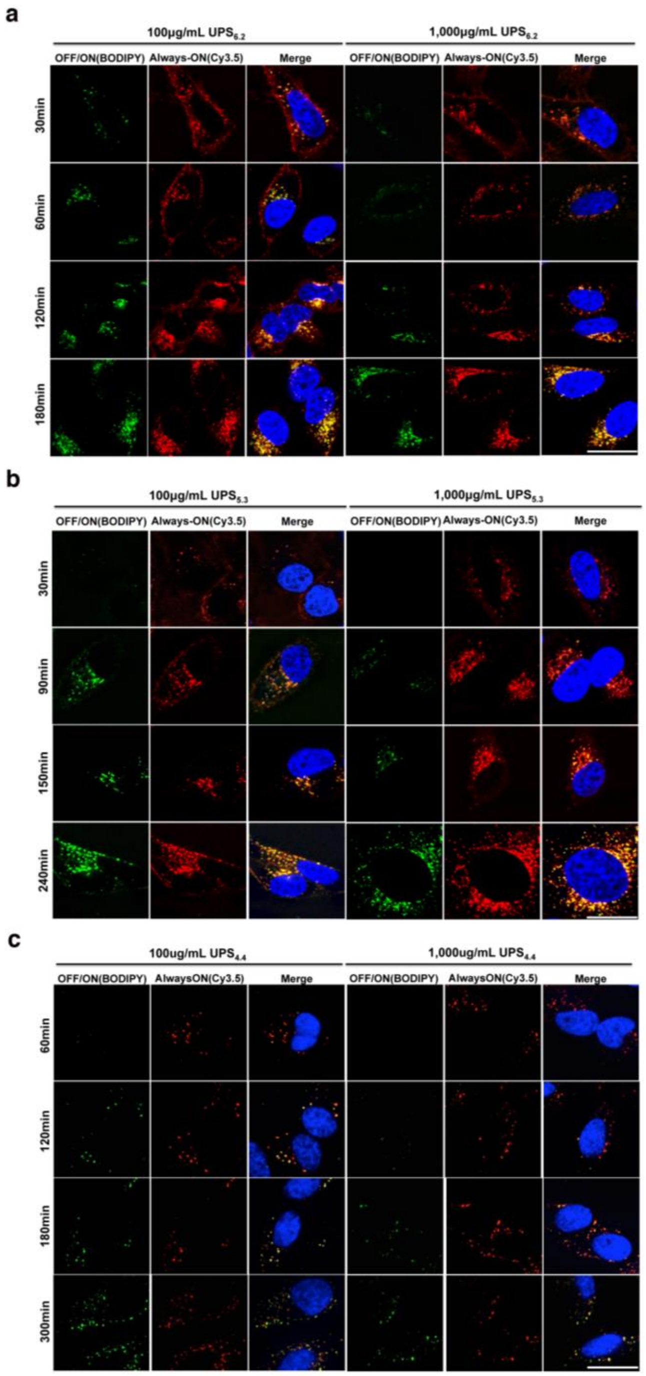

Supplementary Figure 6. Buffering endocytic organelles of HeLa cells with UPS nanoparticles. Representative images of HeLa cells at the indicated time points following a 5 min exposure to low dose (100 μg/mL) and high dose (1,000 μg/mL) Always-ON (Cy3.5) /OFF-ON (BODIPY) $UPS_{6.2}$ (**a**), $UPS_{5.3}$ (**b**) and $UPS_{4.4}$ (**c**). Nuclei were stained blue with Hoechst. Scale bar = 10 μm.

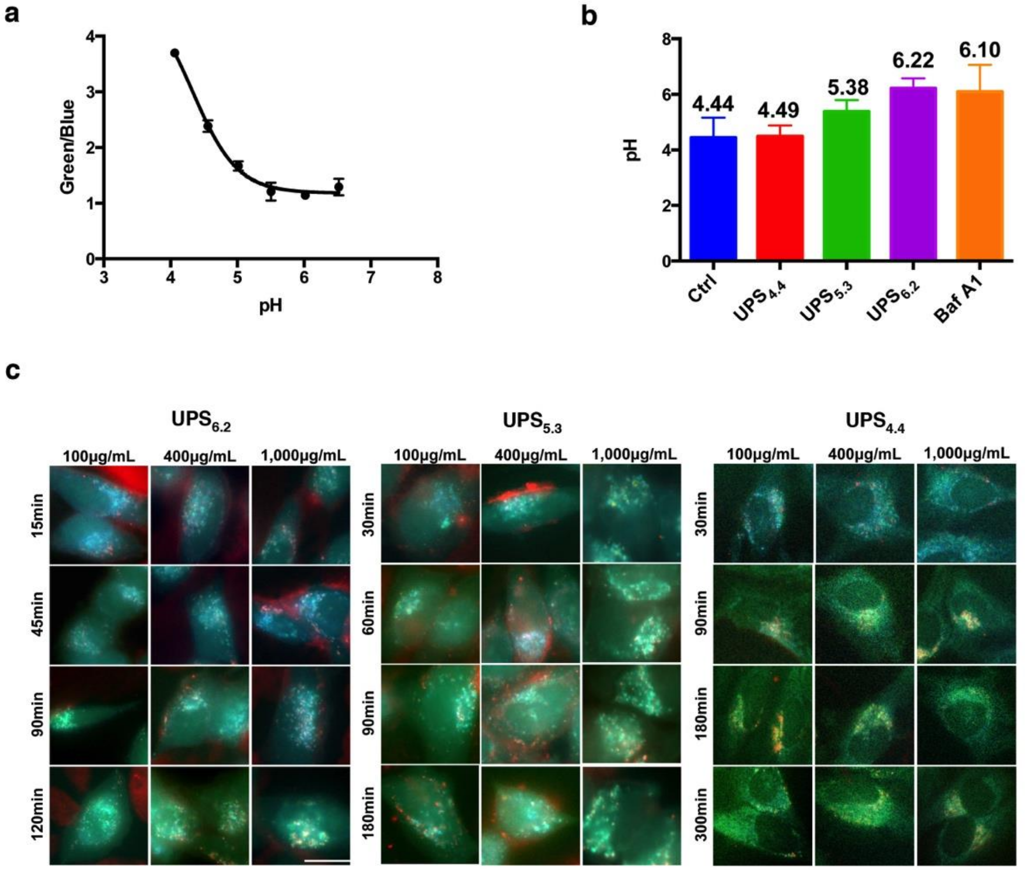

Supplementary Figure 7. ***In situ*** **measurement of luminal pH in HeLa cells.** (**a**) The ratio of fluorescence intensity from DAPI (Blue) and FITC (Green) emission filters as a function of lysosomal luminal pH in HeLa cells. Cells were pretreated with 25 □g/mL Lysosensor Yellow/Blue DND 160 for 5 min. The curve in (**a)** was fit to a Boltzmann Sigmoid function, with R^2=0.9947. (**b**) *In situ* pH measurement of endo/lysosomes treated with 400 µg/mL $UPS_{4.4}$, $UPS_{5.3}$, $UPS_{6.2}$ and 200 nM Bafilomycin A1 (an inhibitor of V-ATPase). Error bars indicate standard deviation, n = 5. (**c**) Representative fluorescent images of cells treated with Lysosensor Yellow/Blue DND 160 and indicated doses of $UPS_{6.2}$, $UPS_{5.3}$ and $UPS_{4.4}$. Scale bar = 10 µm. Images taken from the DAPI channel were pseudo-colored as blue, the images from the FITC channel were pseudo-colored as green, and the images from UPS nanoparticles (Cy5) were pseudo-colored as red.

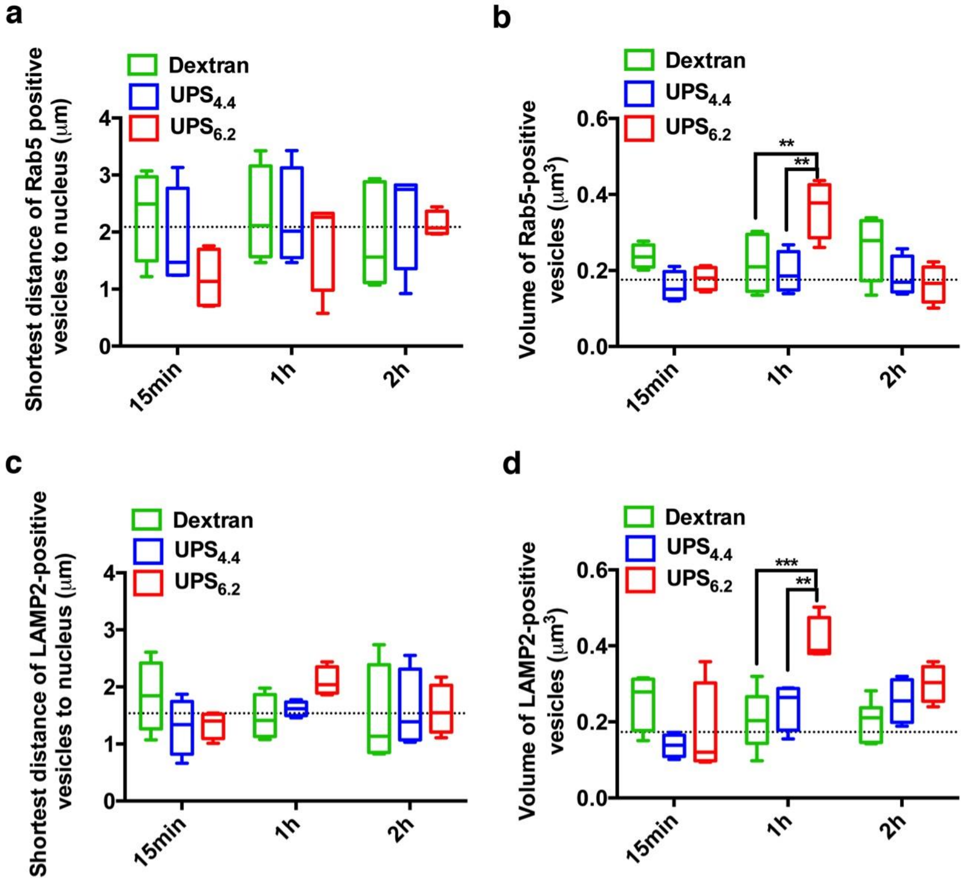

Supplementary Figure 8. Intracellular distribution and organellar volumes of endosomes and lysosomes. The shortest distance of a Rab5- (**a**) or LAMP2- (**c**) positive vesicle to the nucleus was measured in cells treated with 500 μg/mL 70 kD dextran-TMR or 1,000 μg/mL $UPS_{6.2}$-Cy5 or $UPS_{4.4}$-Cy5. The corresponding volumes of the vesicles were as shown in (**b**) and (**d**). The median distance and volume was used for each cell (n= 4-6), $\alpha = 0.05$, ***$p<0.001$, **$p<0.01$. Two-way ANOVA and Sidak's multiple comparison tests were performed to assess the statistical significance.

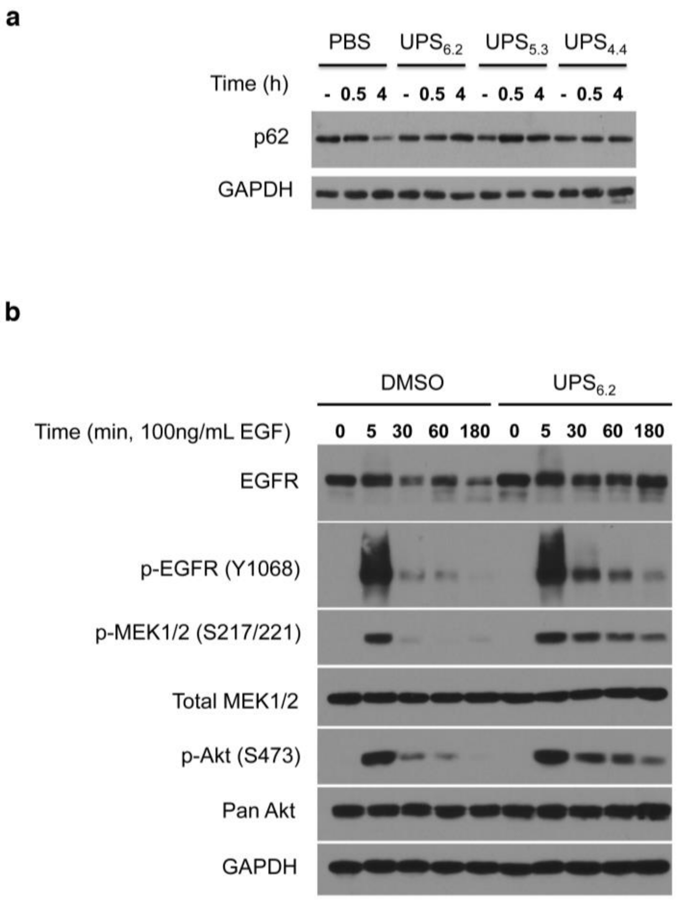

Supplementary Figure 9. Delayed degradation of p62 and EGFR, and prolonged EGF-induced signaling. (**a**) p62 protein accumulation was evaluated by immunoblot with or without 1,000 μg/mL $UPS_{6.2}$, $UPS_{5.3}$ and $UPS_{4.4}$ treatment after serum-starvation up to 4 hours in HeLa cells. (**b**) HeLa cells were serum-starved for 4 hours and stimulated with 100 ng/mL EGF (with or without 1,000 μg/mL $UPS_{6.2}$). Whole cell lysates were collected at indicated time points and analyzed for the indicated proteins.

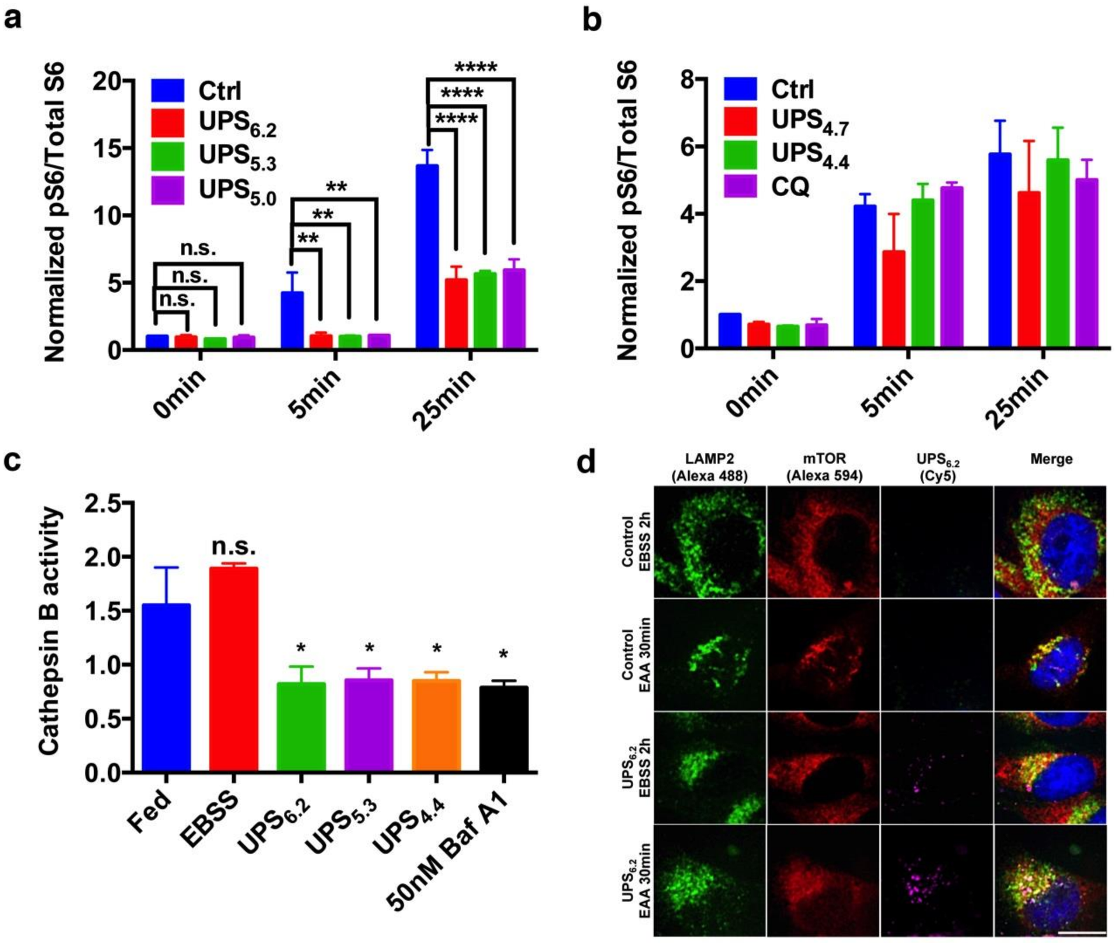

Supplementary Figure 10. mTORC1 signal quantitation and cathepsin B activity upon UPS exposure. (**a**)-(**b**) Quantitative analysis of phosphorylated S6 protein normalized by its total protein levels in **Fig. 4a and b**. Statistic difference between control (water) and UPS treated groups at each time point was detected by two-way ANOVA and Dunnett's multiple comparison test, $\alpha = 0.05$, $^{**}p<0.01$, $^{****}p<0.0001$ or not significant (n.s.). The statistical differences between control and UPS treated groups were not significant at any time point in **b**. Error bars indicate standard deviation, $n = 3$. (**c**) Cathepsin B activity was measured in response to the indicated treatments ($n = 2$). Statistical difference between 'Fed' and all the other groups was detected by one-way ANOVA and Dunnett's multiple comparison test, $\alpha = 0.05$, $^{*}p<0.05$ or not significant (n.s.). (d) Immunofluorescent images of cells stained with mTOR and LAMP2 with or without 1,000 μg/mL $UPS_{6.2}$ under nutrient-deprived (for 2 hours) and nutrient-replete (for 30 minutes) conditions. Scale bar = 10 μm.

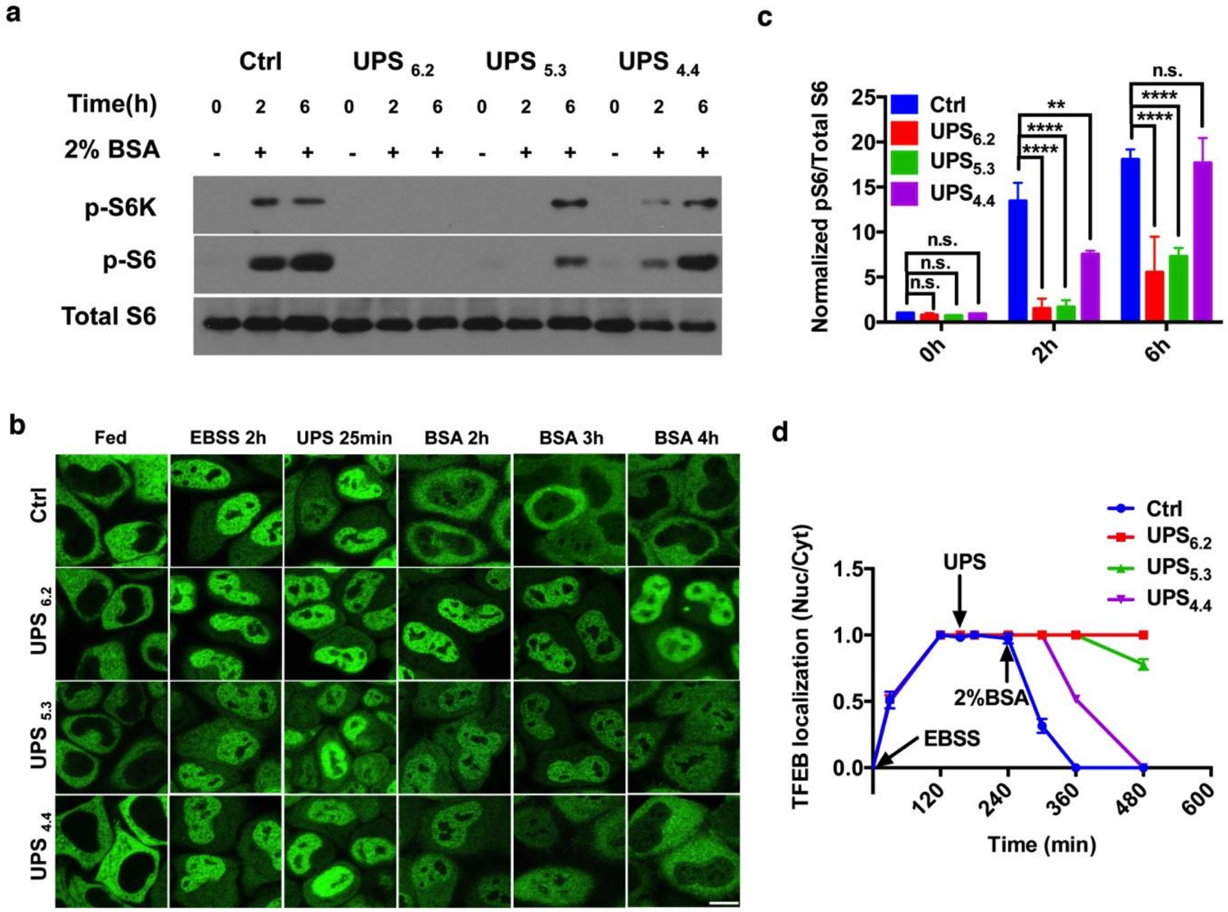

Supplementary Figure 11. Albumin-dependent mTORC1 pathway activation is inhibited by $UPS_{4.4}$. (**a**) HeLa cells were deprived of nutrients for 2 h followed by BSA uptake (2%) in the presence or absence of the indicated UPS nanoparticles (1,000 μg/mL). Accumulation of the indicated phosphoproteins was monitored by immunobot of whole cell lysates. (**b**) Nuclear/cytosolic distribution of GFP-tagged TFEB was monitored in response to the indicated conditions. (**c**) Quantitative analysis of phosphorylated S6 protein normalized by its total protein levels in (**a**). Error bars indicate standard deviation, n = 3. Statistic difference between control and UPS treated groups at each time point was detected by two-way ANOVA and Dunnett's multiple comparison test, α = 0.05, **p<0.01, ****p<0.0001 or not significant (n.s.). (**d**) Quantitative analysis of the location of TFEB in the results shown in (**b**) (in the cytosol = 0, in the nucleus = 1). The error bars represent standard deviation. Scale bar = 10 μm.

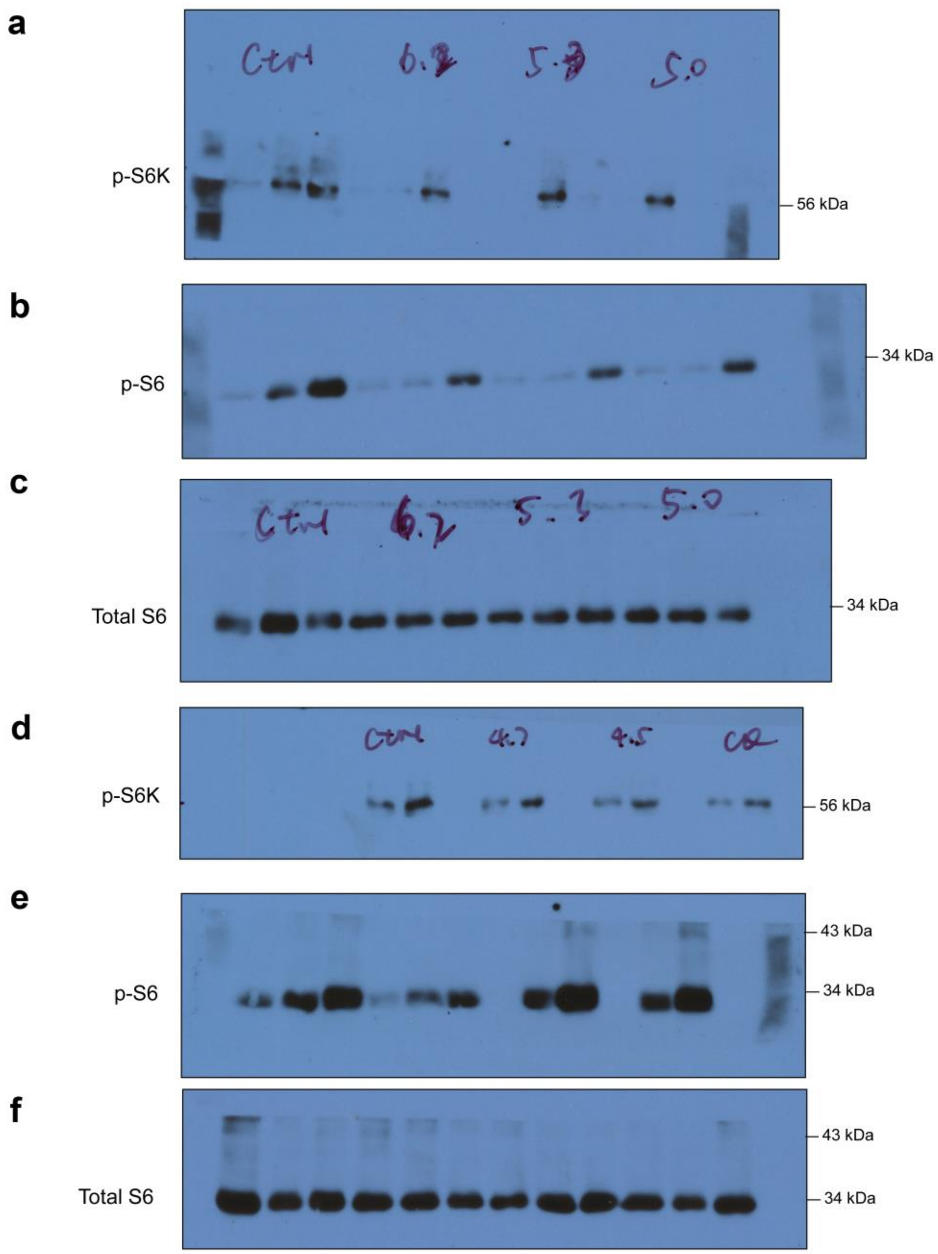

Supplementary Figure 12. Full blots corresponding to the main paper. The full blots shown from panel **a** to **c** and **d** to **e** correspond to the portions of blots in **Fig. 4a** and **Fig. 4b** in the same order.

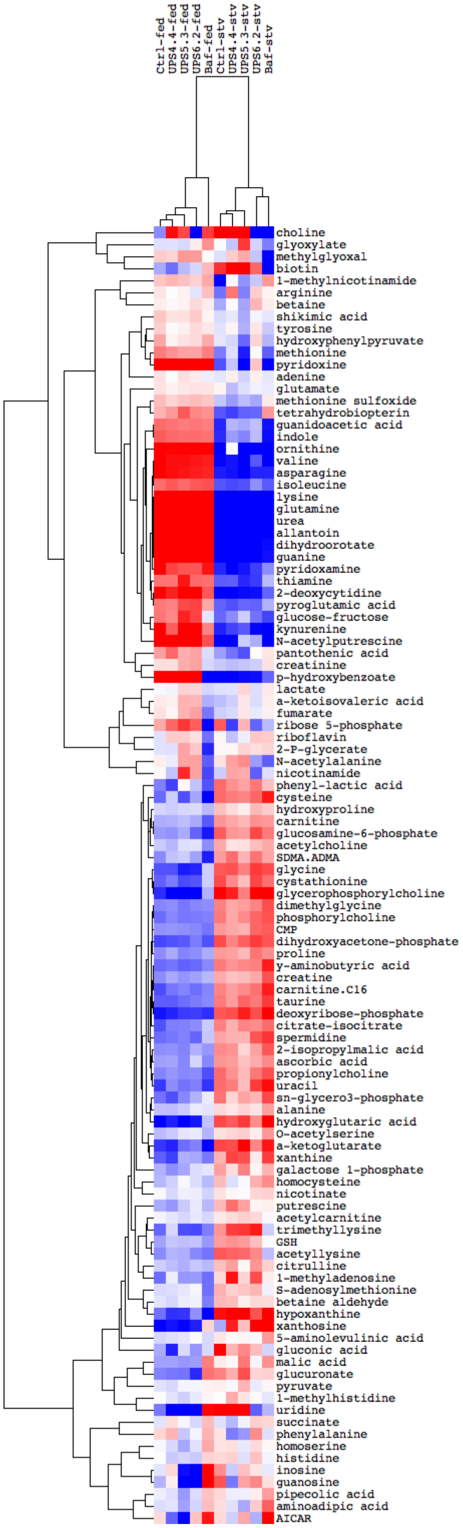

Supplementary Figure 13. An unsupervised hierarchical clustering of metabolites treated with UPS nanoparticles or baf A1 under fed and starved conditions. Average linkage method was used to generate the dendrogram.

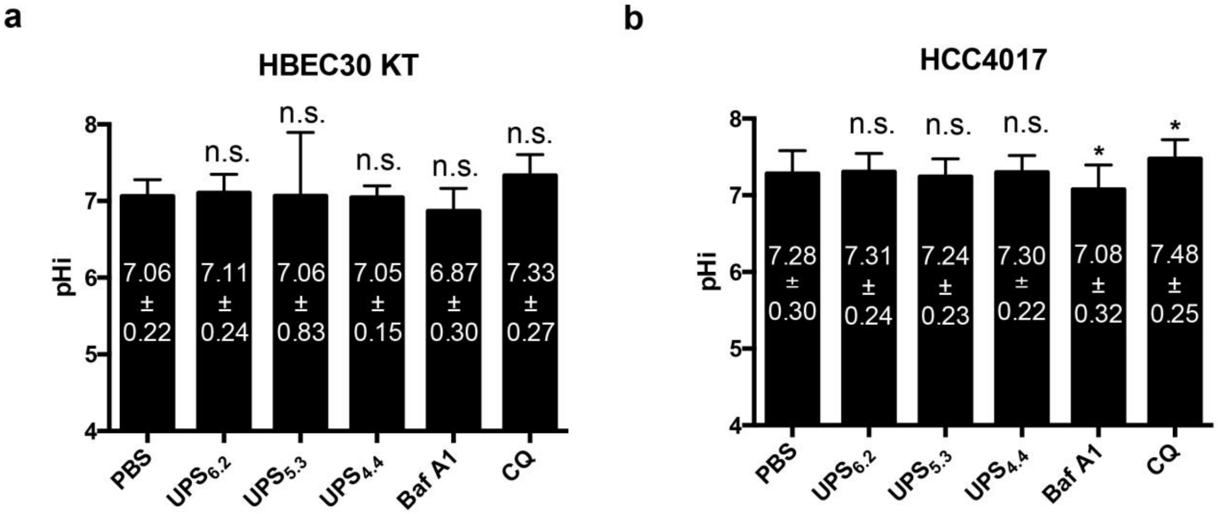

Supplementary Figure 14. Measurement of intracellular pH (pHi). pHi of HBEC30 KT (**a**) and HCC4017 (**b**) cells was measured 24 hours after exposure to 1,000 μg/mL $UPS_{6.2}$, $UPS_{5.3}$, $UPS_{4.4}$, 50 nM baf A1, 1 μM chloroquine (CQ) or PBS using BCECF AM (2',7'-Bis-(2-Carboxyethyl)-5-(and-6)-Carboxyfluorescein, Acetoxymethyl Ester). One-way ANOVA and Dunnett's multiple comparisons test were used to assess statistical significance of observed differences between different treatments and the PBS control group, $\alpha = 0.05$, *$p<0.05$. An average of 40 cells from two independent experiments were assessed for each condition in both cell lines.

Supplementary Table 1. Chemical compositions and physical properties of UPS nanoparticles.

	Composition[a]	D_h (nm)[b]	PDI[b]	ξ (mV)[c]	pK_a[d]	pH_t[e]
**$UPS_{4.4}$**	$P(D5A_{80})$	47.5±3.0	0.13±0.01	-1.1±0.2	4.35	4.39
**$UPS_{4.7}$**	$P(DBA_{28}\text{-}D5A_{52})$	62.4±2.9	0.08±0.01	-0.5±0.1	4.65	4.71
**$UPS_{5.0}$**	$P(DBA_{56}\text{-}D5A_{24})$	54.6±1.2	0.10±0.01	-1.3±0.4	4.93	5.02
**$UPS_{5.3}$**	$P(DBA_{80})$	42.3±2.6	0.12±0.02	-0.7±0.1	5.31	5.32
**$UPS_{5.6}$**	$P(DPA_{30}\text{-}DBA_{50})$	49.8±2.6	0.11±0.01	-2.1±0.4	5.58	5.61
**$UPS_{5.9}$**	$P(DPA_{60}\text{-}DBA_{20})$	49.2±1.3	0.11±0.01	-0.9±0.1	5.89	5.91
**$UPS_{6.2}$**	$P(DPA_{80})$	44.3±1.2	0.10±0.01	-1.6±1.8	6.19	6.22
**$UPS_{6.5}$**	$P(DEA_{21}\text{-}DPA_{79})$	42.0±1.3	0.12±0.02	-0.9±0.6	6.45	6.50
**$UPS_{6.8}$**	$P(DEA_{39}\text{-}DPA_{61})$	35.2±1.3	0.11±0.01	-1.4±0.6	6.77	6.79
**$UPS_{7.1}$**	$P(DEA_{58}\text{-}DPA_{42})$	32.7±1.3	0.13±0.01	-0.9±1.1	7.05	7.08

[a]Only the composition of the PR segment is shown. The subscripts indicate the number of repeating unit for each monomer. [b]The hydrodynamic diameter (D_h) and polydispersity index (PDI) were analyzed by dynamic light scattering analysis. [c]Surface electrostatic potential (ξ) of the UPS nanoparticles was analyzed by the Zeta Sizer. [d]The apparent pK_a values for UPS nanoparticles were measured by pH titration experiments in the presence of 150 mM NaCl. [e]The transition pH (pH_t) was measured from Cy5-conjugated UPS nanoparticles based on fluorescence intensity.

Supplementary Table 2. Quantification of acidification rates of endocytic organelles by the UPS nanoparticles.

	$[UPS]_{med}$ (μg/mL)	D_h/ξ (nm/mV)[a]	No. UPS/cell ($\times 10^2$)[b]	Plateau pH (mean±SD)	t_p (min)[c]	Rate ($\times 10^2$/sec)[d]
**$UPS_{6.2}$**	100	44.3±1.2/ -1.6±1.8	4.7	n.d.	n.d.	n/a
	400		17	6.2±0.1	53	1.7
	1,000		24	6.2±0.1	86	1.5
**$UPS_{5.3}$**	100	42.3±2.6/ -0.7±0.1	4.6	n.d.	n.d.	n/a
	400		17	5.4±0.1	48	1.9
	1,000		24	5.3±0.1	68	1.9
**$UPS_{4.4}$**	100	47.5±3.0/ -1.1±0.2	4.4	n.d.	n.d.	n/a
	400		17	4.5±0.1	67	1.4
	1,000		27	4.4±0.1	81	1.8

[a]Hydrodynamic diameter (D_h) and zeta potential (ξ) were measured in the PBS buffer at pH 7.4. [b]Calculated based on 800 copolymer chains per micelle. [c]t_p is measured as the time interval where the pH was buffered at the plateau value. [d]The rate of proton accumulating in each endocytic organelle.

Supplementary Methods

Syntheses of dye-conjugated PEO-*b*-(P(R_1-*r*-R_2)) block copolymers

Aminoethyl methacrylate (AMA) was used for the conjugation of dyes. Three primary amino groups were introduced into each polymer chain by controlling the feeding ratio of AMA monomer to the initiator (molar ratio = 3). After synthesis, PEO-*b*-(PR-*r*-AMA) (10 mg) was dissolved in 2 mL DMF. Then the Dye-NHS ester (1.5 equivalences for Dye-NHS) was added. After overnight reaction, the copolymers were purified by preparative gel permeation chromatography (PLgel Prep 10μm 10^3Å, 300×25mm column by varian, THF as eluent at 5 mL/min) to remove the free dye molecules. The resulting copolymers were lyophilized and kept at -20 °C for storage. The only difference for the syntheses of block copolymers for always-ON/OFF-ON UPS nanoparticles is that three AMA groups were introduced into a polymer chain for BODIPY conjugation, while one AMA group was introduced for Cy3.5 conjugation.

Preparation and characterization of UPS nanoparticle micelles

In a typical procedure, 10 mg UPS polymer was dissolved in 500 μL THF (without dye conjugation) or methanol (with dye-conjugation). For always-on/OFF-ON UPS nanoparticles, BODIPY-conjugated polymer and Cy3.5-conjugated polymer was mixed with the indicated weight ratio (**Supplementary Fig. S5**) to determine the best combination that yields high ON/OFF ratio in BODIPY channel and stable always-on signal in Cy3.5 channel. The solution was added to 10 mL Milli-Q water drop by drop. Four to five filtrations through a micro-ultrafiltration system (<100 kDa, Amicon Ultra filter units, Millipore) were used to remove the organic solvent. The aqueous solution of UPS nanoparticles was sterilized with a 0.22 μm filter unit (Millex-GP syringe filter unit, Millipore). Transmission electron microscopy (TEM, JEOL 1200 EX model, Tokyo, Japan) was used to examine micelle size and morphology. Dynamic light scattering (DLS, Malvern Nano-ZS model, He-Ne laser, λ= 633 nm) was used to

determine the hydrodynamic diameter (D_h) of 100 μg/mL micelle PBS solutions. The presented data were averaged from five independent measurements. The zeta-potential was measured using a folded capillary cell (Malvern Instruments, Herrenberg, Germany). The presented data were averaged from three independent measurements.

Quantitation of cellular uptake of UPS nanoparticles

HeLa cells (1×10^6 per well) were seeded in 6-well tissue culture dishes. After 12 to 16 h, the cells were exposed to $UPS_{6.2}$-TMR, $UPS_{5.3}$-TMR or $UPS_{4.4}$-TMR for 5 min in serum free DMEM, and then washed three times with PBS. Following an additional 2h incubation in DMEM + 10% FBS, the UPS nanoparticles were extracted from cells with methanol. UPS nanoparticle micelles disassociate into unimers in methanol. A Hitachi fluorometer (F-7500 model) was used to determine RFU of the UPS-TMR unimer solutions at 570 nm. The dose of internalized UPS nanoparticles was calculated from the RFU and a standard curve of the UPS-TMR solutions.

Cytochrome C escape assay

The method was adapted from Lin, M.L. et al[1]. HeLa cells (1×10^5 per well) were seeded in a 96-well plate (Corning). After 24 h, the cells were exposed to 200 μg/mL $UPS_{6.2}$, $UPS_{5.3}$, $UPS_{4.4}$, or 32 μg/mL 25 kD branched PEI (positive control) with or without 3 mg/mL cytochrome C for 5 min in serum free DMEM. No treatment and cytochrome c alone were used as negative controls. Then cells were washed with PBS twice and incubated in DMEM+10%FBS for another 5 hours before a CellTiter-Glo® Luminescent Cell Viability Assay was used to determine the viability of the cells.

Hemolysis assay

The assay was adapted from Bignami's method[2]. Whole mouse blood (2 mL) was added

to 12 mL 0.9% NaCl solution, and was centrifuged at 1000 rpm for 15min. The supernatant was disregarded and 0.9% NaCl solution was added to wash the precipitate at least 3 times until the supernatant become clear. Red blood cell suspension (2%) was prepared in 0.9% NaCl solution. Distilled H_2O (500 □L, positive control), 0.9% NaCl (negative control), or 400 μg/mL UPS nanoparticles in 0.9% NaCl solution were added to 500 □L of red blood cell suspension. The mixed solution was incubated at 37°C for 3 hours, then centrifuged at 1000 rpm for 3min. Supernatant of each sample was read on a Shimadzu UV-Vis spectrophotometer (UV-1800 model) at 570nm.

Metabolomic analysis

HeLa cells were grown in 100 mm dishes until 80% confluent, and separated into nutrient replete and nutrient depleted groups. The medium for cells in the nutrient deplete group was changed to EBSS before being washed with saline twice. Then 200 or 400 μg/mL $UPS_{4.4}$ (final concentration) or same volume of water (as control, each condition contains 6 replicates) was added to both groups and was left for overnight. In a separate cohort of experiments, 1,000 μg/mL $UPS_{6.2}$, $UPS_{5.3}$, $UPS_{4.4}$ or 100 nM baf A1 or water (each condition contains 4 replicates) was added to nutrient replete and nutrient depleted cells and was incubated overnight. Following this, cells were washed twice with ice-cold saline, then overlaid with 500 μL of cold methanol/water (50/50, v/v). Cells were transferred to an Eppendorf tube and subjected to three freeze-thaw cycles. After vigorous vortexing, the debris was pelleted by centrifugation at 16,000 × g and 4°C for 15 min. Pellets were used for protein quantitation (BCA Protein Assay Kit, Thermo). The supernatant was transferred to a new tube and evaporated to dryness using a SpeedVac concentrator (Thermo Savant, Holbrook, NY). Metabolites were reconstituted in 100 μL of 0.03% formic acid in analytical-grade water, vortex-mixed and centrifuged to remove debris. Thereafter, the supernatant was transferred to a HPLC vial for the metabolomics study.

Targeted metabolite profiling was performed using a liquid chromatography-mass

spectrometry/mass spectrometry (LC/MS/MS) approach. Separation was achieved on a Phenomenex Synergi Polar-RP HPLC column (150 × 2 mm, 4 µm, 80 Å) using a Nexera Ultra High Performance Liquid Chromatograph (UHPLC) system (Shimadzu Corporation, Kyoto, Japan). The mobile phases employed were 0.03% formic acid in water (A) and 0.03% formic acid in acetonitrile (B). The gradient program was as follows: 0-3 min, 100% A; 3-15 min, 100% - 0% A; 15-21 min, 0% A; 21-21.1 min, 0% - 100% A; 21.1-30 min, 100% A. The column was maintained at 35°C and the samples kept in the autosampler at 4°C. The flow rate was 0.5 mL/min, and injection volume 10 µL. The mass spectrometer was an AB QTRAP 5500 (Applied Biosystems SCIEX, Foster City, CA) with electrospray ionization (ESI) source in multiple reaction monitoring (MRM) mode. Sample analysis was performed in positive/negative switching mode. Declustering potential (DP) and collision energy (CE) were optimized for each metabolite by direct infusion of reference standards using a syringe pump prior to sample analysis. The MRM MS/MS detector conditions were set as follows: curtain gas 30 psi; ion spray voltages 5000 V (positive) and -1500 V (negative); temperature 650°C; ion source gas 150 psi; ion source gas 250 psi; interface heater on; entrance potential 10 V. In total, 69 water-soluble endogenous metabolites were confidently detected above the baseline set by cell-free samples. Dwell time for each transition was set at 3 msec. Cell samples were analyzed in a randomized order, and MRM data was acquired using Analyst 1.6.1 software (Applied Biosystems SCIEX, Foster City, CA).

Chromatogram review and peak area integration were performed using MultiQuant software version 2.1 (Applied Biosystems SCIEX, Foster City, CA). Although the numbers of cells were similar and each sample was processed identically and randomly, the peak area for each detected metabolite was normalized against the protein content of that sample to correct any variations introduced from sample handling through instrument analysis. The normalized area values were used as variables for the multivariate and univariate statistical data analysis. The chromatographically co-eluted metabolites with shared MRM transitions were shown in a

grouped format, i.e., leucine/isoleucine. All multivariate analyses and modeling on the normalized data were carried out using SIMCA-P (version 13.0.1, Umetrics, Umeå, Sweden).

An unsupervised hierarchical clustering was performed with Gene Cluster 3.0 software. The raw data was firstly normalized to total ion current and was log-transformed and mean-centered. Euclidean distance and average-linkage method were used to generate the dendrograms.

Supplementary References

1. Lin, M.L. et al. Selective suicide of cross-presenting CD8+ dendritic cells by cytochrome c injection shows functional heterogeneity within this subset. *Proc. Natl. Acad. Sci. U. S. A.* **105**, 3029-3034 (2008).
2. Bignami, G.S. A rapid and sensitive hemolysis neutralization assay for palytoxin. *Toxicon* **31**, 817-820 (1993).
